# Site-Specific Phosphorylation of VEGFR2 Is Mediated by Receptor Trafficking: Insights from a Computational Model

**DOI:** 10.1371/journal.pcbi.1004158

**Published:** 2015-06-12

**Authors:** Lindsay Wendel Clegg, Feilim Mac Gabhann

**Affiliations:** 1 Institute for Computational Medicine and Department of Biomedical Engineering, Johns Hopkins University, Baltimore, Maryland, United States of America; 2 Department of Materials Science and Engineering, Johns Hopkins University, Baltimore, Maryland, United States of America; Northeastern University, UNITED STATES

## Abstract

Matrix-binding isoforms and non-matrix-binding isoforms of vascular endothelial growth factor (VEGF) are both capable of stimulating vascular remodeling, but the resulting blood vessel networks are structurally and functionally different. Here, we develop and validate a computational model of the binding of soluble and immobilized ligands to VEGF receptor 2 (VEGFR2), the endosomal trafficking of VEGFR2, and site-specific VEGFR2 tyrosine phosphorylation to study differences in induced signaling between these VEGF isoforms. In capturing essential features of VEGFR2 signaling and trafficking, our model suggests that VEGFR2 trafficking parameters are largely consistent across multiple endothelial cell lines. Simulations demonstrate distinct localization of VEGFR2 phosphorylated on Y1175 and Y1214. This is the first model to clearly show that differences in site-specific VEGFR2 activation when stimulated with immobilized VEGF compared to soluble VEGF can be accounted for by altered trafficking of VEGFR2 without an intrinsic difference in receptor activation. The model predicts that Neuropilin-1 can induce differences in the surface-to-internal distribution of VEGFR2. Simulations also show that ligated VEGFR2 and phosphorylated VEGFR2 levels diverge over time following stimulation. Using this model, we identify multiple key levers that alter how VEGF binding to VEGFR2 results in different coordinated patterns of multiple downstream signaling pathways. Specifically, simulations predict that VEGF immobilization, interactions with Neuropilin-1, perturbations of VEGFR2 trafficking, and changes in expression or activity of phosphatases acting on VEGFR2 all affect the magnitude, duration, and relative strength of VEGFR2 phosphorylation on tyrosines 1175 and 1214, and they do so predictably within our single consistent model framework.

## Author Summary

Vascular endothelial growth factor (VEGF) is an important regulator of blood vessel growth. To date, therapies attempting to harness the VEGF system to promote blood vessel growth (e.g. for wound healing or ischemic disease) have achieved only limited success. To improve VEGF-based therapies, we need to better understand how VEGF promotes development of functional blood vessels. We have developed a computational model of VEGF binding to the receptor VEGFR2, trafficking of VEGFR2 through endosomal compartments in the cell, and activation of VEGFR2 on several tyrosine residues. The pattern of tyrosines activated on VEGFR2 influences cell behavior, promoting cell survival, proliferation, or migration. The combination of these cues influences the diameter of vessels, degree of branching, and leakiness of the resultant vessel network. Our model shows that changes in VEGFR2 trafficking as a result of VEGF immobilization to the extracellular matrix are sufficient to describe observed changes in the pattern of VEGFR2 activation compared to stimulation with purely soluble VEGF. This model can be used to predict how VEGF immobilization, interactions with co-receptors or proteins that deactivate VEGFR2, and changes to VEGFR2 trafficking can be tuned to promote development of functional blood vessel networks for tissue engineering applications.

## Introduction

Members of the vascular endothelial growth factor (VEGF) family are critical regulators of angiogenesis and are implicated as cause or as potential therapy in over 70 diseases, including ischemic diseases of the heart and brain and many cancers. To date, only limited success has been achieved in promoting development of functional vascular networks for tissue engineering [1–3], regeneration [4], and wound healing [5–7]. To harness the VEGF family for tissue vascularization, we must improve our understanding of the mechanisms by which the mode of presentation of VEGF to endothelial cells alters endothelial cell response.

Some VEGF isoforms can bind to proteins and proteoglycans in the extracellular matrix as well as to their cognate cell surface receptors. This matrix-bound VEGF was previously thought to represent a relatively inert pool of sequestered VEGF held in reserve until proteolytic release. Recent work has demonstrated that matrix-bound VEGF can directly ligate and activate VEGF receptors [8–10], and that VEGF and platelet-derived growth factor (PDGF) engineered to have increased affinity for the extracellular matrix promote wound healing and angiogenesis better than the wild-type growth factors [11]. Computational models of VEGF transport predict that the amount of matrix-bound VEGF in normal human tissue (e.g. skeletal muscle) is 30 to 100-fold higher than the amount of free (soluble, unbound) VEGF [12, 13]. However, almost all *in vitro* studies of VEGF receptor signaling have examined only soluble presentation of VEGF. Better mechanistic understanding of how VEGF immobilization alters VEGF receptor 2 (VEGFR2) signaling (and the resulting cellular behavior) will greatly improve our ability to design VEGF-based therapies and to pattern cues for vascular networks in tissue engineering applications.

Multiple isoforms of VEGFA (herein referred to as VEGF) exist, the most common in humans being VEGF_121_, VEGF_165_, and VEGF_189_ (VEGF_120_, VEGF_164_, and VEGF_188_ in mice) [14]. VEGF_165_ and VEGF_189_ include basic heparin-binding domains through which they can bind to extracellular matrix (ECM) proteins such as fibronectin and collagen, and also heparin [15–19]. Tissues express distinct ratios of VEGF isoforms, possibly inducing tissue-specific vascular architecture [20]. Mouse tumors expressing only VEGF_188_ or modified protease-resistant VEGF isoforms exhibit dense, highly branched networks of small diameter blood vessels [21, 22]. In contrast, tissues or tumors secreting primarily VEGF_120/121_ (purely soluble) exhibit wide, tortuous vessels with low branching density and high permeability [22, 23].

**Fig 1:**
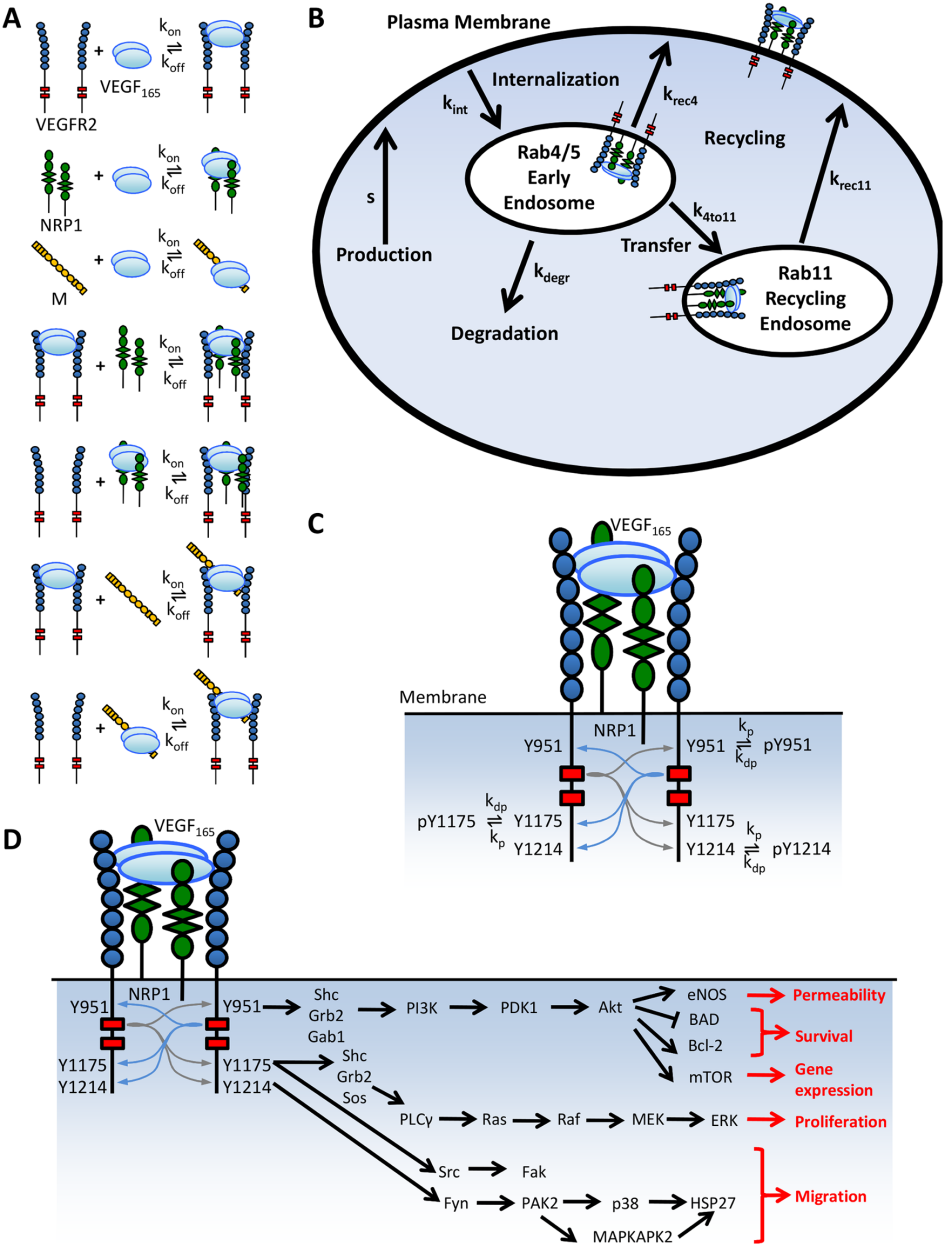
Model schematics. A. Biochemical Reactions. Interactions between VEGF, VEGFR2, NRP1, and an extracellular matrix proteoglycan (M) are summarized. VEGF can bind VEGFR2, NRP1, and M. NRP1 and M cannot be present in the same complex (as they bind to the same surface of VEGF), and VEGFR2 and NRP1 cannot form a complex without VEGF. B. Trafficking Pathways. Surface molecular complexes can be internalized with rate constant k_intn_. Rab4/5-resident molecular complexes can be degraded (rate constant k_degr_), recycled (rate constant k_rec4_), or transferred to the Rab11 compartment (rate constant k_4to11_). Rab11 endosome-resident complexes are recycled with rate constant k_rec11_. New surface receptors are produced at rate s. C. Phosphorylation Reactions. Intracellular tyrosine residues Y951, Y1175, and Y1214 are phosphorylated and dephosphorylated independently. D. Overview of signaling pathways and cellular behaviors downstream of tyrosine residues Y951, Y1175, and Y1214 on VEGFR2.

There are 3 receptor tyrosine kinases (RTKs) for the VEGF ligands. We focus here on VEGFR2, the RTK most strongly associated with VEGF-induced angiogenesis. VEGF, a constitutive dimer, binds and dimerizes two VEGFR2 monomers, resulting in receptor autophosphorylation of multiple intracellular tyrosines. Each phosphotyrosine recruits distinct sets of adaptor proteins, leading to distinct downstream signaling [24, 25] (Fig 1D). Generally, phosphorylation on tyrosine 951 (Y951) promotes Akt activation (via PI3K) and cell survival [1, 26]. Phosphorylation of Y1175 leads to activation of ERK1/2 (via PLCγ) and proliferation, as well as activation of Akt [27, 28], while phosphorylation of Y1214 leads to activation of p38 MAPK and migration [1, 29, 30].

The isoform-specific coreceptor Neuropilin-1 (NRP1) does not bind directly to VEGFR2; NRP1-binding VEGF isoforms bridge VEGFR2 and NRP1 to form a ternary complex [31]. NRP1 increases the effective affinity of VEGF for VEGFR2 [32], as well as influencing VEGFR2 trafficking [33, 34]. NRP1 may have other VEGF-induced, VEGFR2-independent signaling capabilities [35, 36], but these effects are excluded from this analysis.

While many aspects of trafficking are conserved across RTK systems [24, 25, 37–39], there are significant differences between the well-studied EGF system and the VEGF system [40, 41]. Similar to other RTKs, VEGFR2 is constantly being produced, internalized, recycled, and degraded in endothelial cells, resulting in continuous turn-over of the receptor population even in the absence of VEGF [42] (Fig 1B). Upon internalization, VEGFR2 is initially found in early endosomes, identified by the marker Rab5. From the early endosome, VEGFR2 can be recycled directly via Rab4-positive endosomes, degraded via Rab7-positive late endosomes, or transferred to Rab11-positive recycling endosomes [40, 43, 44]. Constitutive recycling of VEGFR2 in endothelial cells occurs primarily through the Rab4 pathway [40, 41], while NRP1 promotes routing of VEGF-VEGFR2 complexes through the Rab11–positive recycling pathway [40, 45]. An increasing body of evidence suggests that trafficking of VEGFR2 regulates receptor phosphorylation, downstream signaling, and the resulting cell behavior [24, 30, 46, 47]. VEGFR2 signaling can be attenuated by receptor degradation or by tyrosine phosphatase-mediated dephosphorylation of intracellular residues [9, 48, 49]. Significant gaps exist in current understanding of phosphatases acting on VEGFR2, but the subcellular localization and tyrosine residue specificity of some phosphatases is known.

Vascularization is critical for the viability of thick tissue-engineered constructs. As such, there is significant interest in immobilizing VEGF in tissue scaffolds or on surfaces. Though a variety of different techniques for immobilization of VEGF have been explored, a clear understanding of the mechanism through which immobilized VEGF results in different cellular signaling and vascular phenotypes than soluble VEGF has not yet emerged. We and others have previously developed models to study the trafficking of VEGFR2 [8, 24, 25] and other growth factor receptors (most notably EGF receptors [50–52]), as well as the impact of growth factor sequestration in the extracellular matrix on distribution and availability to cells [53–55]. However, these models have only considered matrix-bound VEGF, VEGFR2 trafficking, and receptor phosphorylation events in a limited way, if at all.

The purpose of this study is to quantitatively connect trafficking and localization of VEGFR2 to tyrosine site-specific VEGFR2 phosphorylation patterns, which have been implicated in regulating signaling downstream of VEGFR2. Specifically, this model was developed to test the hypothesis that control of site-specific VEGFR2 dephosphorylation by receptor trafficking is sufficient to explain experimental observations following stimulation of endothelial cells with soluble or immobilized VEGF. We also study the effect of NRP1 expression on VEGFR2 trafficking and phosphorylation.

## Materials and Methods

### Computational Model Overview

We developed a model to simulate *in vitro* experiments in which monolayers of endothelial cells are exposed to exogenous soluble or immobilized VEGF. The model uses a large set of coupled, nonlinear, deterministic, ordinary differential equations to simulate key biochemical reactions (Fig 1A), trafficking processes (Fig 1B), and phosphorylation reactions (Fig 1C). The model uses experimentally-derived kinetics (Tables 1–3) and incorporates geometry and initial concentrations specific to the experimental protocols being simulated (Table 4). In the model, we assume that VEGF and VEGFR2 are pre-dimerized, though we have examined the implications of including dimerization reactions in the past [56]. Both soluble VEGF (V) and ECM-bound or immobilized VEGF (V·M) can bind to VEGFR2 (R2). V·M can dissociate and reassociate. NRP1 (N1) can bind to V or V·R2, but not to M·V·R2, as N1 and M bind to the same heparin-binding domain on VEGF. We exclude for now the possibility of one NRP1 and one M binding to opposite sides of the same VEGF dimer simultaneously, though this assumption was tested. These reactions occur in every model compartment (described below) in which the corresponding species are present.

**Table 1:** Model parameters for biochemical reactions.

Species 1	Species 2	k_on_	k_off_	K_D_	Reference
**External**					
V	M	Varies	Varies	Varies	Fit
**Surface**					
V	R2	1.0 x 10^7^ M^-1^s^-1^	1.0 x 10^-3^ s^-1^	1.0 x 10^-10^ M	[57]
M	V·R2	4.2 x 10^5^ M^-1^s^-1^	1.0 x 10^-2^ s^-1^	2.4 x 10^-8^ M	Assumed same as V·M
V·M	R2	1.0 x 10^7^ M^-1^s^-1^	1.0 x 10^-3^ s^-1^	1.0 x 10^-10^ M	Assumed same as V·R2
V	N1	4.8 x 10^4^ M^-1^s^-1^	1.0 x 10^-4^ s^-1^	2.1 x 10^-9^ M	[58]
N1	V·R2	3.1 x 10^13^ (mol/cm^2^)^-1^s^-1^	1.0 x 10^-3^ s^-1^	3.2 x 10^-17^ mol/cm^2^	[57]
V·N1	R2	1.0 x 10^14^ (mol/cm^2^)^-1^s^-1^	1.0 x 10^-3^ s^-1^	1.0 x 10^-17^ mol/cm^2^	[57]
V·M	N1	0 M^-1^s^-1^	-	-	Assumed
V·M·R2	N1	0 M^-1^s^-1^	-	-	Assumed
M	V·N1	0 M^-1^s^-1^	-	-	Assumed
**Internal (same in Rab4/5 and Rab11 endosomes)** ^**a**^					
V	R2	1.0 x 10^7^ M^-1^s^-1^	1.0 x 10^-3^ s^-1^	1.0 x 10^-10^ M	[57]
V	N1	4.8 x 10^4^ M^-1^s^-1^	1.0 x 10^-4^ s^-1^	2.1 x 10^-9^ M	[58]
N1	V·R2	3.1 x 10^13^ (mol/cm^2^)^-1^s^-1^	1.0 x 10^-3^ s^-1^	3.2 x 10^-17^ mol/cm^2^	[57]
V·N1	R2	1.0 x 10^14^ (mol/cm^2^)^-1^s^-1^	1.0 x 10^-3^ s^-1^	1.0 x 10^-17^ mol/cm^2^	[57]

^a^ All rates for internal reactions were assumed to be the same as on the cell surface. Unit conversion was required (not shown) for k_on_ and K_D_ in internal compartments (from M^-1^s^-1^ to (#/cm^2^)^-1^s^-1^ for k_on_ and from M to #/cm^2^ for K_D_) using the total volume of the specified endosomal compartment per unit surface area (See S2 Table- not assumed to be spherical). Note that all rates are effective rates; diffusion considerations are lumped into these effective rates.

**Table 2:** Model parameters for trafficking.

Parameter	Species	Value (s^-1^)	Reference
k_int_	R2	2.6 x 10^-3^	[24, 25]
	V·R2	3.12 x 10^-2^	[24, 25]
	M·V·R2	0	Assumed
	V	0	Assumed
	N1	2.6 x 10^-3^	Assumed same as R2
	V·N1	2.6 x 10^-3^	Assumed same as N1
	V·N1·R2	3.12 x 10^-2^	Assumed same as V·R2
k_rec4_	**R2_rab45_**	**3.8 x 10^-3^**	Fit
	V·R2_rab45_	3.8 x 10^-3^	Held equal to k_rec4_ for R2 _rab45_ during fitting
	V_rab45_	0	Assumed
	N1_rab45_	3.8 x 10^-5^	Held equal to 1/100 * k_rec4_ for R2 _rab45_ during fitting
	V·N1_rab45_	3.8 x 10^-5^	Held equal to k_rec4_ for N1 _rab45_ during fitting
	V·N1·R2_rab45_	3.8 x 10^-5^	Held equal to k_rec4_ for N1 _rab45_ during fitting
k_rec11_	R2_rab11_	1.4 x 10^-4^	Held equal to 1/100 * k_rec11_ for N1_rab11_ during fitting
	V·R2_rab11_	1.4 x 104	Held equal to 1/100 * k_rec11_ for N1_rab11_ during fitting
	V_rab11_	0	Assumed
	**N1_rab11_**	**1.4 x 10^-2^**	Fit
	V·N1_rab11_	1.4 x 10^-2^	Held equal to k_rec4_ for N1_rab11_ during fitting
	V·N1·R2_rab11_	1.4 x 10^-2^	Held equal to k_rec4_ for N1_rab11_ during fitting
k_4to11_	R2_rab45_	1.0 x 10^-5^	Assumed
	V·R2_rab45_	1.0 x 10^-5^	Assumed
	V_rab45_	0	Assumed
	**N1_rab45_**	**1.9 x 10^-2^**	Fit
	V·N1_rab45_	1.9 x 10^-2^	Held equal to k_4to11_ for N1_rab45_ during fitting
	V·N1·R2_rab45_	1.9 x 10^-2^	Held equal to k_4to11_ for N1_rab45_ during fitting
k_degr_	R2_rab45_	3.6 x 10^-6^	Held equal to 1/10 * k_degr_ for V·R2_rab45_ during fitting
	**V·R2_rab45_**	**3.6 x 10^-5^**	Fit
	V_rab45_	1.2 x 10^-2^	Assumed
	**N1_rab45_**	**1.6 x 10^-4^**	Fit
	V·N1_rab45_	1.6 x 10^-4^	Held equal to k_degr_ for N1_rab45_ during fitting
	**V·N1·R2_rab45_**	**6.8 x 10^-4^**	Fit
s	R2	Calculated	Calculated (Units: #/(cm^2^s))
	N1	Calculated	Calculated (Units: #/(cm^2^s))

**Bold**: Value fit directly in this study

**Table 3:** Model parameters for VEGFR2 phosphorylation.

Species	k_p_ (All Residues)	k_dp,Y951_ (s^-1^)	k_dp,Y1175_ (s^-1^)	k_dp,Y1214_ (s^-1^)
R2	0	30	30	30
R2_rab45_	0	30	30	30
R2_rab11_	0	30	30	30
V·R2	1	**0.043**	**4.98**	**1.06**
V·R2_rab45_	1	**75.0**	**0.00972**	**0.0307**
V·R2_rab11_	1	30	30	30
V· N1·R2	1	6	5	1
V· N1·R2_rab45_	1	15	0.01	6
V· N1·R2_rab11_	1	30	30	30

All units s^-1^: **Bold = fit**. References: See Methods.

**Table 4:** Initial conditions and parameters that vary by study.

Parameter	Trafficking Study [40]	2011 Presentation Study [8]	2010 Presentation Study [29]	Other Simulations
Cell Type	PAEC	HUVEC	HUVEC	HUVEC
**Initial Conditions**				
[V]	50 ng/mL	2 ng/mL	200 ng/mL	Varies
[M]	-	1500 ng/mL	3 mg/mL	3 mg/mL
[R2]_surf_	37,400/108,000 per cell	6,000 per cell	6,000 per cell	6,000 per cell
[N1]_surf_	0/113,000 per cell	35,000 per cell	35,000 per cell	35,000 per cell
**Geometry**				
Total Surface Area	1 cm^2^	3 cm^2^ ^b^	1 cm^2^	1 cm^2^
Solution/Matrix Depth^a^	0.5 cm	0.05 cm (Vb)/ 0.5 cm (Vs)	0.5 cm	0.5 cm
**Trafficking Parameters (factor change from values in Table 2)**				
k_int_(V·R2)	-	-	/6	-^c^
k_degr_	-	x 2.4	x 2.4	x 2.4
**V**·**M Binding Parameters**				
k_off,V·M_	-	3.3 x 10^-3^ s^-1^ (Ve) / 1.1 x 10^-3^ s^-1^ (Vc)	1.0 x 10^-2^ s^-1^	1.0 x 10^-2^ s^-1^
k_on,V·M_	-	4.2 x 10^5^ M^-1^s^-1^	4.0 x 10^3^ M^-1^s^-1^	4.0 x 10^3^ M^-1^s^-1^
k_D,V·M_	-	7.9 x 10^-9^ M (Ve)^d^ / 2.6 x 10^-9^ M (Vc)	2.5 x 10^-6^ M	2.5 x 10^-6^ M

^a^ Calculated from information in [8] on surface densities and VEGF concentration, and used to calculate [M].

^b^ Tuned so total surface Vs·R2 + internal VEGF matches data in [8].

^c^ The original k_int_ value was used for [V] < 50 ng/mL (trafficking study [40], 2011 presentation study [8], Anderson et al. 2011 Validation study [59], Mellberg et al. [60]), as well as for all model predictions. k_int_/6 was used for [V] > 50 ng/mL (2010 presentation study [29], Martino et al. Validation study [61], and Mattila et al. [62]). In cases where [V] = 50 ng/mL, the value that resulted in better fits was used.

^d^ References: [12, 63]

### Trafficking Processes

The model includes five compartments: extracellular (media and matrix); cell surface; early endosomes (combining Rab4- and Rab5-positive endosomes); recycling endosomes (Rab11-positive); and degraded (late/Rab7-positive endosomes and lysosomes). The concentration of soluble or immobilized VEGF in the extracellular compartment is assumed to be uniform, but not constant. Newly synthesized VEGFR2 and NRP1 are inserted into the cell surface compartment at a constant rate, resulting in constant surface VEGFR2 and NRP1 populations in the absence of VEGF. The initial quantity of VEGFR2 and NRP1 in each endosomal compartment was set based on the trafficking parameters to obtain a steady distribution in the absence of VEGF. R2, V·R2, N1, V·N1, and V·N1·R2 are internalized from the surface to the early endosome compartment. M·V·R2 is not internalized in our model; we assume it is anchored to the gel or surface to which VEGF is immobilized. All receptor complexes in the Rab4/5 compartment can be recycled directly, degraded, or trafficked to the Rab11 compartment for recycling. We assume that free (non-receptor-bound) VEGF in Rab4/5 endosomes does not recycle or travel to Rab11 endosomes; quantitative analysis showed that these processes were negligible compared to degradation of free VEGF. We assumed that no degradation occurs from the Rab11 compartment, as Rab11 endosomes have an outward-directed motor (for recycling). The trafficking processes are summarized in Fig 1B. Examples of the equations describing the biochemical reactions and trafficking of each species are given below. The complete set of biochemical and trafficking reactions (not including all phosphorylation states of VEGFR2) can be found in the Supplemental Information.

Extracellular Molecular Complexes:

$$\begin{array}{c} \frac{d\left[V\right]}{dt}=-k_{on,V\cdot M}\left[V\right]\left[M\right]+k_{off,V\cdot M}\left[V\cdot M\right]-k_{on,V\cdot R2}\left[V\right]\left[R2\right]+k_{off,V\cdot R2}\left[V\cdot R2\right]\;-k_{on,V\cdot N1}\left[V\right]\left[N1\right]\\ +k_{off,V\cdot N1}\left[V\cdot N1\right] \end{array}$$

Cell Surface Molecular Complexes:

$$\begin{array}{c} \;\frac{d\left[V\cdot R2\right]}{dt}=k_{on,V\cdot R2}\left[V\right]\left[R2\right]-k_{off,V\cdot R2}\left[V\cdot R2\right]-k_{on,M\cdot \left(V\cdot R2\right)}\left[M\right]\left[V\cdot R2\right]\\ +k_{off,M\cdot \left(V\cdot R2\right)}\left[M\cdot V\cdot R2\right]-k_{on,\left(V\cdot R2\right)\cdot N1}\left[V\cdot R2\right]\left[N1\right]\\ +k_{off,\left(V\cdot R2\right)\cdot N1}\left[V\cdot N1\cdot R2\right]-k_{intn,V\cdot R2}\left[V\cdot R2\right]+\;k_{rec4,V\cdot R2}\left[{\left(V\cdot R2\right)}_{rab45}\right]\\ +\;k_{rec11,V\cdot R2}\left[{\left(V\cdot R2\right)}_{rab11}\right] \end{array}$$

Rab4/5 Molecular Complexes:

$$\begin{array}{c} \frac{d\left[R2_{rab45}\right]}{dt}=-k_{on,V\cdot R2_{rab45}}\left[V_{rab45}\right]\left[R2_{rab45}\right]+k_{off,V\cdot R2_{rab45}}\left[{\left(V\cdot R2\right)}_{rab45}\right]\\ -k_{on,\left(V\cdot N1\right)\cdot R2_{rab45}}\left[{\left(V\cdot N1\right)}_{rab45}\right]\left[R2_{rab45}\right]+k_{off\left(V\cdot N1\right)\cdot R2_{rab45}}\left[{\left(V\cdot N1\cdot R2\right)}_{rab45}\right]\\ +k_{intn,R2}\left[R2\right]-\;k_{rec4,R2_{rab45}}\left[R2_{rab45}\right]-\;k_{4to11,R2_{rab45}}\left[R2_{rab45}\right]-\;k_{degr,R2_{rab45}}\left[R2_{rab45}\right] \end{array}$$

### Phosphorylation Reactions

Phosphorylation and dephosphorylation of ligated VEGFR2 (V·R2) and free VEGFR2 (R2) take place on the cell surface and in the endosomes. We assume that the intrinsic phosphorylation and dephosphorylation rates for V·R2 are the same whether VEGF is immobilized or soluble. We also assume that NRP1 does not affect the intrinsic phosphorylation rates of VEGFR2, though it does increase the affinity of VEGF for VEGFR2. While some information about the subcellular locations and tyrosine specificities of phosphatases targeting VEGFR2 is available, there is insufficient information to develop detailed explicit phosphatase models. Thus, we assume first order phosphorylation and dephosphorylation kinetics, effectively assuming that the relevant phosphatases are present in excess. The phosphorylation and dephosphorylation rates for each tyrosine residue examined (Y951, Y1175, and Y1214) can be independent in the model, and vary with the ligation status and subcellular localization of VEGFR2. Thus, we do not assume that VEGFR2 is automatically phosphorylated upon binding of VEGF or dephosphorylated upon unbinding of VEGF; these are still first-order reactions. We assume that the trafficking rates in the model are controlled by the ligation status of VEGFR2, not by its phosphorylation state. VEGFR2 internalization has been shown to be regulated by phosphorylation of Y1054/Y1059 [64]. However, as these are considered activation tyrosine residues, we assume that VEGFR2 ligation is a surrogate for pY1054/59, as done in previous models [24, 25]. We assume that phosphorylation of all tyrosine sites on VEGFR2 is lost upon degradation, but that phosphorylation and ligation patterns are not directly changed by other trafficking processes.

Given the three tyrosine sites being considered, there are eight possible phosphorylation patterns for VEGFR2: R2 (no phosphorylation), R2_pY951_, R2_pY1175_, R2_pY1214_, R2_pY951-pY1175_, R2_pY951-pY1214_, R2_pY1175-pY1214_, and R2_pY951-pY1175-pY1214_. The same patterns are possible for V·R2, M·V·R2, V·N1·R2, R2_rab45_, V·R2_rab45_, V·N1·R2_rab45_, R2_rab11_, V·R2_rab11_, and V·N1·R2_rab11_. We assume that all newly produced VEGFR2 is completely unphosphorylated. These reactions are shown schematically for V·R2 on the cell surface in Fig 1C. A sample equation describing the complete set of biochemical reactions, trafficking processes, and phosphorylation events that affect the population of surface R2_pY1175_ is given below:

$$\begin{array}{c} \frac{d\left[R2_{pY1175}\right]}{dt}=-k_{on,V\cdot R2}\left[V\right]\left[R2_{pY1175}\right]+k_{off,V\cdot R2}\left[V\cdot R2_{pY1175}\right]-k_{on,(M\cdot V)\cdot R2}\left[M\cdot V\right]\left[R2_{pY1175}\right]\\ +k_{off,(M\cdot V)\cdot R2}\left[M\cdot V\cdot R2_{pY1175}\right]-k_{intn,R2_{pY1175}}\left[R2_{pY1175}\right]+\;k_{rec4,R2_{rab45,pY1175}}\left[R2_{rab45,pY1175}\right]\\ +\;k_{rec11,R2_{rab11,pY1175}}\left[R2_{rab11,pY1175}\right]+k_{p,Y1175,R2}\left[R2\right]-k_{dp,Y1175,R2_{pY1175}}\left[R2_{pY1175}\right]\\ -k_{p,Y951,R2_{pY1175}}\left[R2_{pY1175}\right]+k_{dp,Y951,R2_{pY951-pY1175}}\left[R2_{pY951-pY1175}\right]\\ -k_{p,Y1214,R2_{pY1175}}\left[R2_{pY1175}\right]+k_{dp,Y1214,R2_{pY1175-pY1214}}\left[R2_{pY1175-pY1214}\right] \end{array}$$

### Model Outputs

**Fig 2:**
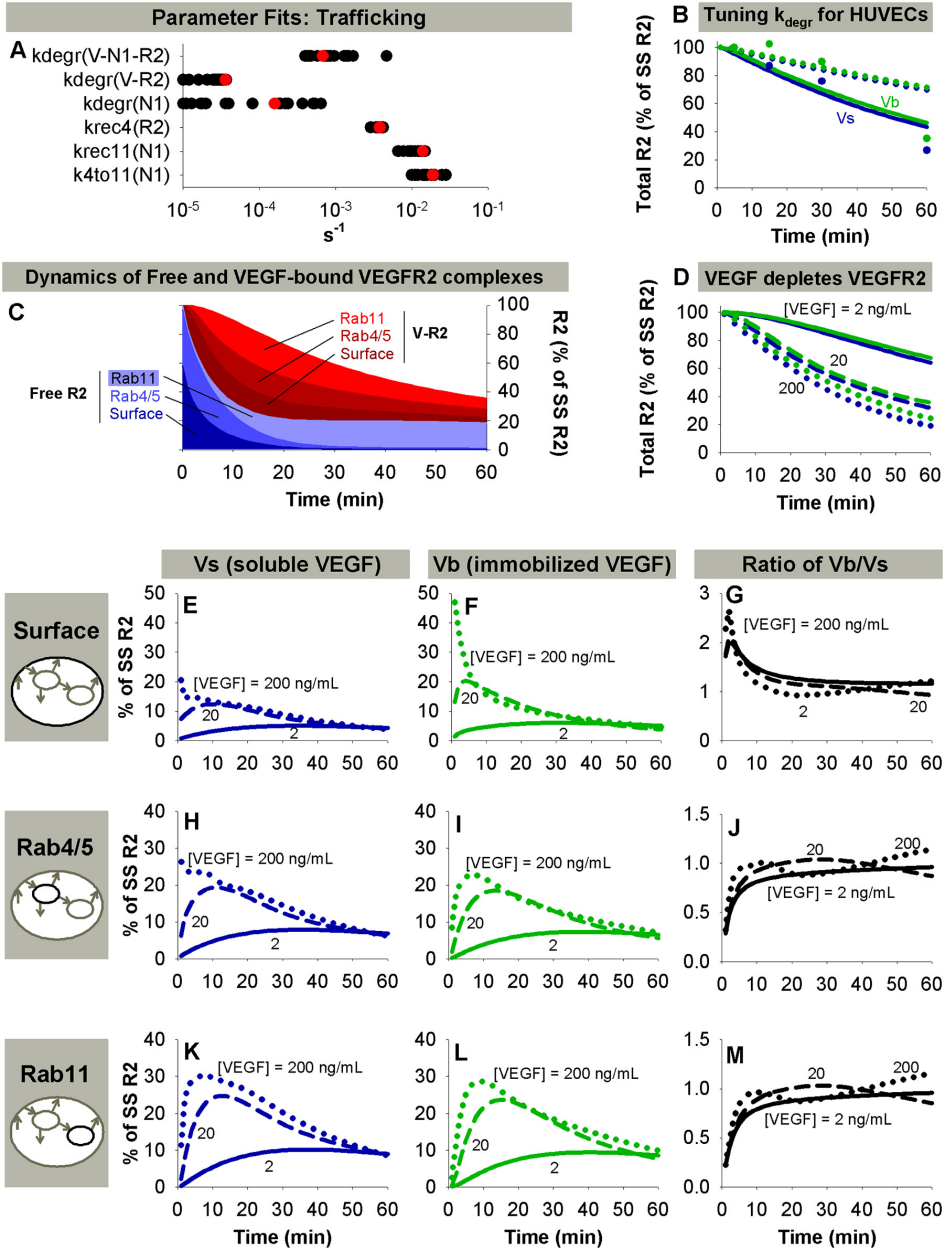
VEGF presentation and trafficking control the distribution of ligated VEGFR2. A. Visualization of fit trafficking parameters. Red dots indicate a representative coherent set of parameters used throughout the rest of this study. (See Methods) B. Tuning of k_degr_ values to match data in 2010 presentation study. Dotted lines: fits using k_degr_ values fit in PAECs; solid lines: fits after k_degr_ values are increased by a factor of 2.4 to minimize the least squared error between simulations and this experimental data. Soluble VEGF (Vs), blue lines; bound VEGF (Vb), green lines. C. Summary of the distribution of VEGF-bound (V·R2) and unbound VEGFR2 (Free R2) over time, shown for Vb. Y-axis is shown in terms of the percentage of the steady-state (no VEGF) total VEGFR2 population. Note that, due to degradation, the total amount of VEGFR2 decreases after addition of VEGF. [V] = 20 ng/mL. D. Decrease in total VEGFR2 upon stimulation with VEGF. Solid line, [V] = 2 ng/mL; dashed line, [V] = 20 ng/mL; dotted line, [V] = 200 ng/mL. E-M. Distribution of V·R2 on the cell surface (E-G), in Rab4/5 endosomes (H-J), and in Rab11 endosomes (K-M) following stimulation with Vs (left column) or Vb (middle column). The right column shows the ratio of the first two columns.

The outputs of this model are the concentrations of each molecule or molecular complex (summarized in S1 Table) over time. The concentrations of certain complexes were combined into lumped quantities of interest, which could be directly compared to experimental data. For example, the model allows prediction of pY951 VEGFR2 (pY951), pY1175, and pY1214 quantities over time under different simulation conditions. These quantities are obtained by summing the concentrations of all VEGFR2 (ligated and free) that are phosphorylated on the given site. These quantities are examined in total, and also partitioned into surface, Rab4/5, and Rab11 components. We assume that the total VEGFR2 phosphorylated on at least one of Y951, Y1175, and Y1214 in the model is a reasonable correlate to total phosphorylated VEGFR2 (pR2) experimental data. As we do not include all tyrosine residues on VEGFR2 in our model, this is expected to underestimate the total percentage of VEGFR2 phosphorylated, but the shape of the resulting curve is expected to be correct. Model outputs were normalized for parameter estimation in the same way as the experimental data to which they were compared. For model predictions, outputs are shown relative to the total VEGFR2 in the system in the absence of VEGF (100%) (see Fig 2C). Note that, due to degradation, the total amount of VEGFR2 in the system after VEGF stimulation is less than 100%.

### Parameter Fitting Overview

Parameters in this model were taken from literature, calculated, assumed, or fit to data from one study of VEGFR2 and NRP1 trafficking [40] (hereafter referred to as the “trafficking study”) and two studies of immobilized VEGF [8, 29] (hereafter referred to as the “2010 presentation study” and the “2011 presentation study,” together the “VEGF presentation studies”). The provenance of each parameter is shown in Tables 1–3. The experimental trafficking study provides the most detailed and complete quantitative data on VEGFR2 and NRP1 trafficking from a single study to date [40]. The VEGF presentation studies provide data on site-specific phosphorylation of VEGFR2 upon exposure to soluble and immobilized VEGF [8, 29], allowing us to study site- and location-specific phosphorylation patterns. These studies have experimental outputs that can be directly compared to model outputs for parameter estimation and validation. We simulated the experimental protocols from these studies by altering the initial conditions and geometry in the model to fit the protocol used in each study, as summarized in Table 4.

The general protocol used to assemble a completely parameterized model was as follows. All biochemical reaction rates, excepting V·M reaction parameters, were taken from literature or previous experimentally-validated models of VEGF_165_, VEGFR2, and NRP1, as summarized in Table 1. The trafficking parameters were fit to data in the trafficking study [40] (soluble VEGF only). The assumptions used to reduce the number of parameters fit are detailed in Table 2 and below. Next, we fixed the dephosphorylation rates for all species in Rab11 endosomes at a sufficiently high value to minimize phosphorylation in this compartment, as pY1175 and pY1212 (mouse equivalent of human Y1214) did not co-localize with Rab11 in the trafficking study. We then took the model, to this point parameterized solely with soluble VEGF data, and applied it to the VEGF presentation studies [8, 29]. The M·V reaction parameters were fit to data in each study. We then fit phosphorylation parameters for the cell surface and Rab4/5 endosomes to data from both of the VEGF presentation studies simultaneously. We fixed the phosphorylation rates and fit the dephosphorylation rates in each compartment, as phosphorylation is assumed to be fast upon ligand binding. We then validated the complete model against data from four independent studies and additional data from the trafficking study [40, 59–62]. This modular approach to parameter fitting was chosen to reduce the number of parameters being fit at each step, and because distinct types of data (cell lines and experimental set-ups) were used for each step. It does not reduce the utility of our model in determining whether trafficking-controlled dephosphorylation of VEGFR2 is sufficient to account for observed trends in experimental data.

### Trafficking Parameters

We fixed the internalization rates in the model using values for free and ligated VEGFR2 estimated for previous models of VEGFR2 internalization, recycling, and degradation [24, 25]. Our goal was to obtain a validated parameter set that gives correct VEGFR2 distributions, rather than validated, identifiable values for each individual parameter. Receptor distribution is controlled by internalization, recycling, and degradation (production rates for receptors are determined using these values). As such, fitting the recycling and degradation rates should be adequate to capture the trafficking dynamics in this system. We assumed relationships between some of the trafficking parameter values for different molecular species (based upon prior knowledge) to reduce the number of parameters to be fit, given the limited and noisy data available (Table 2). This set was sufficient to capture the trafficking processes of interest in this analysis. Each assumption was relaxed during parameter fitting, to verify that it was reasonable. The listed set of assumptions resulted in the parameter set that best described the data in the trafficking study. The trafficking study examined colocalization of NRP1 and VEGFR2 with over-expressed, fluorescently-tagged Rab4, Rab5, Rab7, and Rab11 in porcine aortic endothelial cells (PAECs) transfected with VEGFR2, NRP1, or both, and exposed to soluble VEGF [40]. Data was also given on total VEGFR2 and NRP1 over time, and total cell-surface NRP1 over time [40]. We included an additional term in the optimization cost function to constrain the percent of total VEGFR2 on the cell surface to approximately 60% in the absence of VEGF, ensuring a biologically reasonably VEGFR2 distribution [41]. For this study, we assumed PAECs transfected with VEGFR2 had 37,400 surface VEGFR2/cell, and cells transfected with VEGFR2 and NRP1 had 108,000 cell surface VEGFR2/cell and 113,000 cell surface NRP1/cell, as previously reported [65].

The experimental data from the trafficking study [40] that was used to fit the trafficking parameters, the weights used on each piece of data in the cost function, and the corresponding simulated values for a representative set of parameters are shown in S4 Table. Data in that study is given for both Rab4- and Rab5-positive endosomes [40]. The maximum of these values was used to fit the data for the Rab4/5 compartment in the model. We fit parameter sets using the relative values of each molecular species in Rab4/5, Rab11, and Rab7 endosomes, normalized so that these three values summed to 100% of the internal population of that receptor. We also tried using the ratio of each quantity in Rab4/5 endosomes to Rab11 endosomes (Number in Rab4/5 endosomes / Number in Rab11 endosomes) to fit the trafficking parameters. This second strategy was considered because we compared Rab7 measures to internal degraded quantities in the model. While species routed for degradation in real cells pass through Rab7 endosomes, degraded species in our model are not removed from the system, and so these quantities increase over time. The resulting parameter sets were similar, and the first approach was pursued.

We fit the trafficking parameters using the Levenberg-Marquardt algorithm, a non-linear least squares optimization routine. Values of all fit parameters were constrained to the range [10^-^ ^5^, 1] s^-1^ to ensure physiologically reasonable parameter estimates. Initial parameter values were pulled randomly from a distribution that is uniform on a log_10_ scale constrained to the range [10^-^ ^4^, 10^-^ ^2^] s^-1^. This initial range resulted in approximately ½ of optimal parameter sets found being accepted, where a parameter set was accepted if the total cost for the set was within 15% of the value for the lowest cost set. From the resulting 23 acceptable parameter sets, a representative parameter set was selected for use in the remainder of this study (shown in Table 2).

The VEGF presentation studies were performed in human umbilical vein endothelial cells (HUVECs) [8, 29]. For all studies using HUVECs, we assumed surface populations of 6,000 VEGFR2/cell and 35,000 NRP1/cell [65]. Due to the differences in receptor numbers between PAECs and HUVECs, and the use of constitutively active, overexpressed Rab proteins in the trafficking study [40], some difference in trafficking parameters between the trafficking and VEGF presentation studies was anticipated. To adjust, the degradation rates for all molecular species were increased by a constant factor (2.4, Table 4), which was determined by minimizing the least square error between model simulations and experimental data in the 2010 presentation study for total VEGFR2 over the time range 0–60 minutes [29].

### Matrix-Binding Parameters

With trafficking parameters set, we next manually fit the VEGF-matrix reaction parameters. The 2011 presentation study immobilized VEGF to a gold-coated slide using a modified heparin linker, using two different protocols to create either an electrostatic or covalent bond between the heparin surface and VEGF [8]. Here, we fixed K_D_ for the electrostatic case to be consistent with literature data on VEGF-heparin affinity [63], and fit k_off,V·M_. For the covalent case, k_off,V·M_ was tuned, also altering K_D,V·M_. After examination of the protocols used, we determined that the measurements of internalized VEGF presented in this study [8] (used to fit k_off,V·M_) likely also contain surface-bound quantities of VEGF. Thus, we compared these measurements to the total internal and soluble surface-bound VEGF quantity in our model. The 2010 presentation study immobilized VEGF in a collagen and fibrinogen gel [29]. VEGF in the collagen gel was assumed to be immobilized primarily through heparin, and the value of k_off,V·M_ was assumed to be 10^-2^ s^-1^. K_D,V·M_ was then tuned to give acceptable fits for phosphorylation data in this study.

### Phosphorylation Parameters

**Fig 3:**
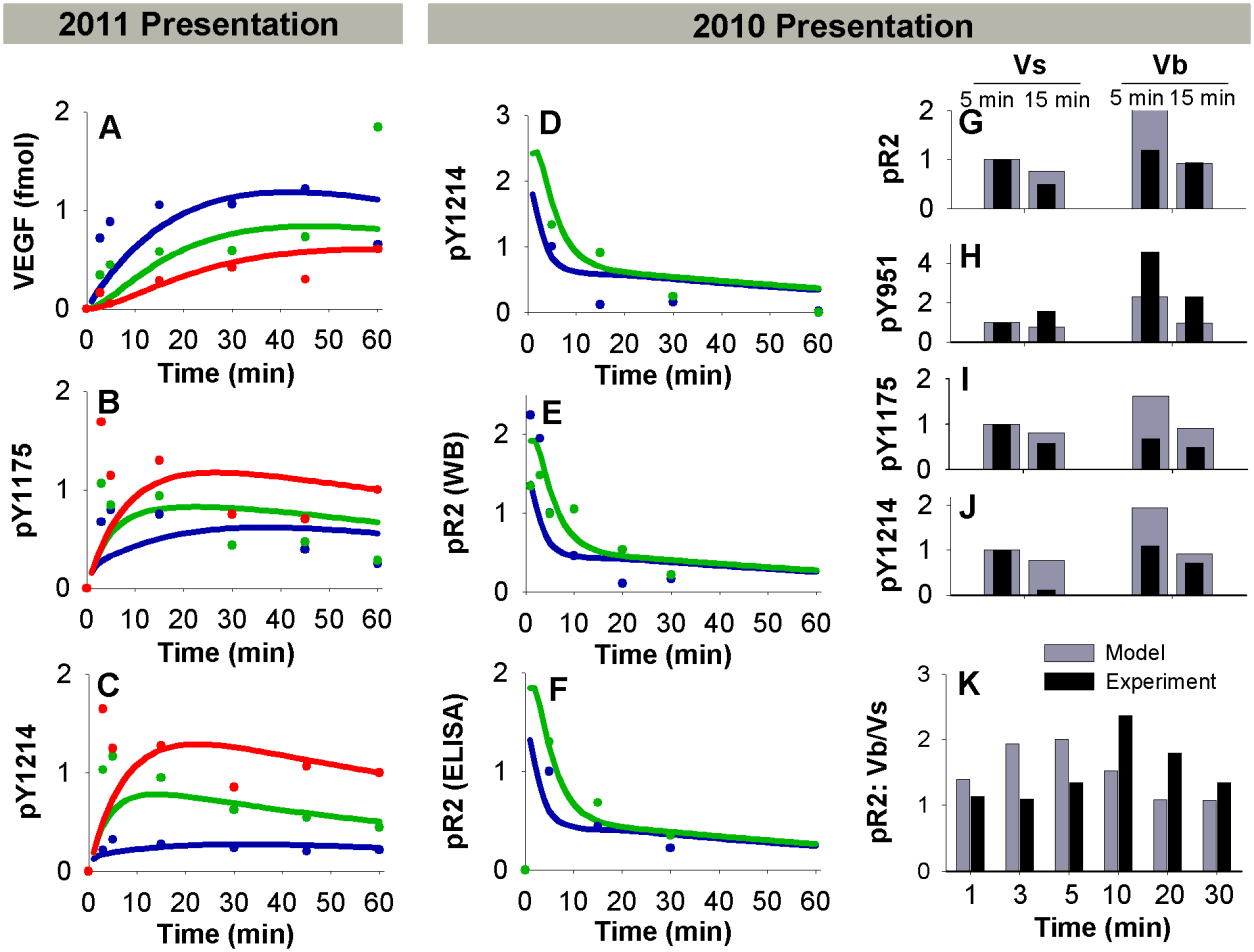
Prediction of VEGFR2 binding and phosphorylation parameters. A. Total surface Vs·R2 + internal VEGF, used to fit k_off,M·V_ for the 2011 presentation study [8]. Lines indicate simulation results, dots indicate experiments. Soluble VEGF (Vs), blue; electrostatic bound VEGF (Ve), green; covalent bound VEGF (Vc), red. B-C. pY1175 and pY1214 data for 2011 presentation study [8], which was used to fit dephosphorylation rate constants, along with H. D-F. Phosphorylation data from the 2010 presentation study [29], which was used to confirm dephosphorylation rate fits. D-E. Western blot data. F. ELISA data. Soluble VEGF, blue; bound VEGF, green. G-J. Additional phosphorylated VEGFR2 (by residue) experimental data from 2010 presentation study with model predictions. K. Ratio of pR2 for Vb and Vs at various times, from 2010 presentation study.

Since phosphorylation is assumed to be a fast process that quickly reaches quasi-static equilibrium upon ligation of VEGFR2, we assumed a constant value of k_p_ for V·R2 for all residues in all compartments, and tuned the dephosphorylation rates. We assume k_p_ is zero for free VEGFR2; thus, free VEGFR2 can transiently maintain its phosphorylation status if VEGF unbinds, but it cannot become phosphorylated without a preceding ligation event. Additionally, we assume a constant high value for k_dp_ for free VEGFR2 in all compartments. Sensitivity studies were performed to confirm that the chosen value was sufficiently high to result in efficient dephosphorylation of VEGFR2 following dissociation of ligand; thus the model predictions were relatively insensitive to this parameter. We also assume that the same constant high k_dp_ value holds for all complexes in Rab11 endosomes, based on qualitative observations in the trafficking study that neither pY1175 nor pY1214 VEGFR2 colocalizes with Rab11 [40]. The remaining k_dp_ values, for Y951, Y1175, and Y1214 on the cell surface and in Rab4/5 endosomes, were fit using the Levenberg-Marquardt algorithm. The dephosphorylation rate constants were fit directly to a portion of the experimental data from the VEGF presentation studies, including Vb/Vs ratios (Fig 3B, 3C and 3H). The fits were then checked against the remaining data from the VEGF presentation studies for reasonableness of fit (Fig 3D–3G, 3I–3K) [8, 29]. When fitting, we excluded early time-points, as our phosphorylation dynamics (controlled by receptor ligation and trafficking) cannot capture these early peaks, similar to models of EGFR [66], rendering optimization ineffective. A model that captures the full complexity of phosphorylation at very early times (<5 min) remains a task for future targeted studies; here we focus on the later time-points, which our model can achieve. Initial values for all parameters were pulled from a wide range [10^-^ ^3^, 100], using a log-uniform distribution, and updated parameters were constrained to the same range. Parameter sets were accepted if the final cost was within 65% of the lowest cost set. Sets were excluded if the predicted number of phosphoVEGFR2 were too low to be realistic (<10 per cell at peak activation); this eliminates unrealistic scenarios and is necessary because the data used to fit is normalized, and so the total phosphorylation levels are not well-constrained. The lowest cost set found was selected as a representative parameter set, and used throughout the remained of this study (shown in Table 3)

### Model Size

The complete model contains 97 molecular species, including all of the considered phosphorylation states for VEGFR2 and all of the locations each protein can exist in the model (surface, Rab4/5 endosomes, Rab11 endosomes, degraded). All biochemical reactions and trafficking processes are assumed to be independent of the phosphorylation state of VEGFR2 in this model. This results in a total of 15 reversible binding reactions that occur across all model compartments, all but one of which were parameterized using data from literature (Table 1). The model has 31 trafficking reactions, with 13 unique trafficking parameters. Two of these were taken from literature, 6 were fit directly, and 5 were assumed or related to other parameter values (Table 2). There are 120 reversible phosphorylation reactions in the model, with 9 unique phosphorylation parameters. Three of these parameters were assumed, and the other 6 were fit directly (Table 3). Including all phosphorylation states of VEGFR2 considered, the model contains a total of 277 reactions, with 52 unique parameters, 13 of which were fit directly, 31 taken from literature, and 8 of which were assumed or calculated based on relationships to other values. Additionally, there are 8 geometric parameters in the model (S2 and S4 Tables), two of which are specific to the experimental set-up of each study (Table 4), and initial concentrations and numbers of receptors per cell for each molecular species (Table 4). While this model is large in size, previous work and simplifying assumptions (discussed in preceding sections) reduce it to a tractable size.

### Validation and Additional Model Simulations

The complete, parameterized model was validated against additional data in the trafficking study and against four independent studies [59, 61]. The additional data in the trafficking study compared colocalization of VEGFR2 with Rab proteins following stimulation with the isoform VEGF_165b_ (V_165b_), which may account for a substantial portion of VEGF_165_ present *in vivo* [67] but lacks the capability to bind NRP1 [68], and VEGF_165a_ (V), which does bind NRP1. The next two validation studies included both soluble and immobilized VEGF. Outputs for the first of these validation studies are pY1175 and pY1214 [59]. The same k_off,V·M_ values were used here as for 2011 presentation study [8]. An assumption that the concentration of immobilized VEGF was lower (by a factor of 3) than that reported for this particular study was necessary to match experimental observations, in particular that pY1175 was lower with immobilized VEGF than with soluble VEGF, and the magnitude of increase in pY1214 with immobilized compared to soluble VEGF. We hypothesize that this reduction in apparent matrix-bound VEGF concentration is due to reduced spatial availability/accessibility of this particular form of VEGF (immobilized on nanoparticles) to endothelial cells. Such a correction was unnecessary for the VEGF presentation studies because the 2011 presentation study presented VEGF in a monolayer and the 2010 presentation study used a high VEGF concentration, so depletion was negligible within the analyzed time-frame (not shown). For the second of these validation studies, the output is total phosphorylated VEGFR2 (pR2) [61]. Here, M·V binding parameters were taken from literature [18]. The initial conditions and geometry were tuned to the experimental set-up, assuming that VEGF and fibronectin (FN) or FN fragments were pre-mixed and allowed to equilibrate before presentation to endothelial cells. We did not change any other model parameters. The third and fourth validation studies examined perturbations to VEGFR2 dephosphorylation [60, 62] using only soluble VEGF. To mimic the experimental perturbations, we altered the relevant dephosphorylation parameters. See Table 4 for a summary of parameters used in additional simulations to probe trafficking and phosphorylation.

### Sensitivity Analysis

We performed a local sensitivity analysis to identify parameters that most strongly affect model outputs. Selected parameters were increased and decreased (one at a time) by a factor of 2, and the absolute value of the percent change in each output was calculated. The values resulting from upward and downward perturbations were averaged. These values were then averaged across a selected set of model outputs. We selected the following model outputs for our sensitivity analysis: pY1175 VEGFR2, pY1214 VEGFR2, and the ratio pY1214/pY1175, at 5, 15, and 30 minutes, for soluble and matrix-bound VEGF at concentrations of 2 ng/mL and 200 ng/mL.

### Model Solution

The set of coupled ordinary differential equations that comprise this model were solved using a 5^th^ order accurate Runge-Kutta scheme with an adaptive step-size. The algorithm was implemented in Fortran on a desktop PC. Simulation run-time was short for all cases.

## Results

### Trafficking Parameter Estimates Appear Consistent across Cell Lines and Studies

Our primary goal was to quantify how changes in trafficking of VEGFR2 lead to altered patterns of site-specific phosphorylation. We first optimized the model trafficking parameters against data on VEGFR2 and NRP1 trafficking in PAECs [40] (S3 Table). The biochemical reaction parameters used are summarized in Table 1, and the initial conditions and geometry used are summarized in the first column of Table 4. The distributions of accepted parameter values (based on many parallel fits) are summarized in Fig 2A and S4 Table. A representative parameter set was selected for use in the model (Fig 2A, red dots). These values are shown in Table 2, along with assumptions made to reduce the number of parameters being fit. Based upon our analysis, we found that assuming a ratio of 1/100 for the relative recycling rates from Rab4/5 and Rab11 endosomes for several molecular species (as indicated in Table 2) produced optimal results. In general, the model captured the relative proportions of a molecule distributed between Rab4/5 and Rab11 endosomes well. The proportion of a species in Rab7 endosomes was typically lower in the experimental data than the degraded quantity in the model, but this was anticipated, as the model does not explicitly represent Rab7 endosomes separately from the degraded compartment. The relative agreement between simulated and experimental values (S3 Table) for this parameter set indicate that the model likely reproduces key elements of the underlying trafficking processes.

Using the obtained trafficking parameters, the model predicts that in the absence of NRP1, internalized V·R2 will be preferentially recycled via the Rab4 pathway or degraded (k_rec4_ > k_degr_ > k_4to11_). Conversely, in the presence of NRP1, internalized V·R2 will be preferentially routed via the Rab11 pathway for recycling (k_4to11_ & k_rec11_ > k_degr_ > k_rec4_). Interestingly, the degradation rate for V·N1·R2 was predicted to be an order of magnitude larger than the degradation rate for V·R2 (Table 2, Fig 2A), suggesting a potential role for NRP1 in regulating VEGFR2 degradation. Our parameter set is also consistent with the Rab11 pathway being the “faster” recycling pathway, accessible only after stimulation with VEGF.

We next compared predictions using these trafficking parameters to data on VEGF presentation in a different endothelial cell type (HUVECs) [8, 29]. The study-specific parameters for these VEGF presentation studies are shown in Table 4. The degradation rates for all species were increased by a constant factor (2.4) to match experimental measurements of total VEGFR2 loss from the 2010 presentation study [29] (Fig 2B). In addition, the internalization rate for V·R2 was decreased slightly for the 2010 presentation study [29]. We hypothesize that this difference in internalization rates is due to the 100-fold difference in VEGF concentration between the experiments, as increasing the numbers of occupied receptors has been shown to decrease the measured internalization rate, likely due to saturation of endocytic pathways [50, 69]. These modifications are summarized in Table 4.

Because obtaining good agreement between the simulated and experimental data in HUVECs did not require many changes in the parameters fit for PAECs, we propose that trafficking may be more consistent across endothelial cell lines than previously thought. Observed differences between cell lines may be the result of changes in absolute and relative receptor densities, along with the difference in degradation rates mentioned above. The obtained trafficking parameter values were also in reasonable agreement with previous estimates [8, 24, 25]. Our estimated degradation rates were lower than previous values, likely because this is the first model to consider dephosphorylation explicitly, allowing for decreases in phosphorylated VEGFR2 without receptor degradation or release of ligand.

The distribution of free and ligated VEGFR2 is predicted to vary as a function of both VEGF concentration and mode of VEGF presentation (Fig 2E–2M, S1 Fig). Prior to stimulation with VEGF, the model predicts that most intracellular VEGFR2 is located in Rab4/5 endosomes (Fig 2C), consistent with experimental observations [41]. Upon stimulation with VEGF, the proportion of VEGFR2 in Rab11 endosomes increases, while total and surface VEGFR2 decrease (Fig 2C). Immobilized VEGF (Vb) results in increased magnitude and duration of V·R2 on the cell surface, and delayed peaks of decreased magnitude for V·R2 in both endosomal compartments compared to soluble VEGF (Vs) (Fig 2E–2M). The magnitude and width of the V·R2 peak was highly dependent upon VEGF concentration in all compartments (Fig 2E–2M). At 30 minutes, the majority of remaining VEGFR2 is predicted to be ligated at VEGF concentrations of 20 ng/mL and higher (Fig 2C, S1 Fig).

### Parameterization of VEGF Release from the Matrix

We next estimated the VEGF-matrix reaction parameters for the VEGF presentation studies. For the 2011 presentation study using a modified heparin linker [8], the equilibrium dissociation constant (K_D_) for VEGF and matrix in this study was taken from literature [12, 63]. The off-rate constant, k_off,V·M_, was estimated to be 3.3 x 10^-3^ s^-1^ in the electrostatic case (Ve), which is within the range reported literature for VEGF and heparin [12, 63] (Fig 3A). For the covalent case (Vc), this value was decreased until a reasonable fit was obtained (Table 4). For the 2010 presentation study, we found that a surprisingly low value of k_off,V·M_ was necessary to fit the experimental release data. This value was too low to allow for sufficient internalization of V·R2 after stimulation with immobilized VEGF to be consistent with experimentally observed data (not shown). We believe therefore that k_off,V·M_ is being underestimated using this release data, as VEGF must both release and diffuse out of the gel to be measured in that assay. Instead, we assumed that VEGF was immobilized in the collagen gel in a heparin-mediated manner, and we used a k_off,V·M_ of 10^-2^ s^-1^. This allowed us to estimate a K_D,V·M_ value for this study (Table 4).

### Parameterization of Phosphorylation Reactions

The final set of parameters we estimated were the dephosphorylation rate constants for Y951, Y1175, and Y1214 on the cell surface and in Rab4/5 endosomes. All other phosphorylation parameters (see Table 3) were fixed as described in the Methods. We fit the dephosphorylation rates to data from the two VEGF presentation studies simultaneously [8, 29], with no study-specific changes in these values. The distribution of accepted parameters values from many fit sets are shown in S2 Fig and summarized in S4 Table. A representative parameter set (S2A Fig, red dots; Table 3) is used in the rest of this study. While a wide range of dephosphorylation parameter values resulted in acceptable fits of the data, the ratios of the surface and Rab4/5 dephosphorylation rate constants for Y1175 and Y1214 were more consistent (S2 Fig). The dephosphorylation rates for 1175 and 1214 were higher on the surface than in Rab4/5 endosomes, while the surface/Rab4/5 ratio was higher for Y1175 than Y1214 in 46 of the 47 accepted parameter sets (S2 Fig). Slightly better fits could be obtained if the parameters were permitted to be study-dependent, or if additional experimental set-up or cell line-specific changes were made; however, we used a consistent parameter set to keep the model as general as possible across multiple experimental set-ups (see Table 3). The fits for these studies are shown in Fig 3A–3K and S2 Fig. In general, important trends in dynamics and relative activation by soluble or matrix-bound VEGF were captured by the model, as were the original experimental values 10 minutes or more after stimulation with VEGF. Additionally, our estimated phosphorylation and dephosphorylation rate constants are consistent with those previously estimated for EGFR [66]. As the estimated dephosphorylation rate constants for Y951 were not well-constrained, and only one set of data was available for pY951, we have limited confidence in the Y951 dephosphorylation rate constants. We therefore focus on VEGFR2 phosphorylated on at least one of Y951, Y1175 and Y1214 (pR2), and VEGFR2 phosphorylated specifically on Y1175 (pY1175) or Y1214 (pY1214).

### Validation of Complete Model

**Fig 4:**
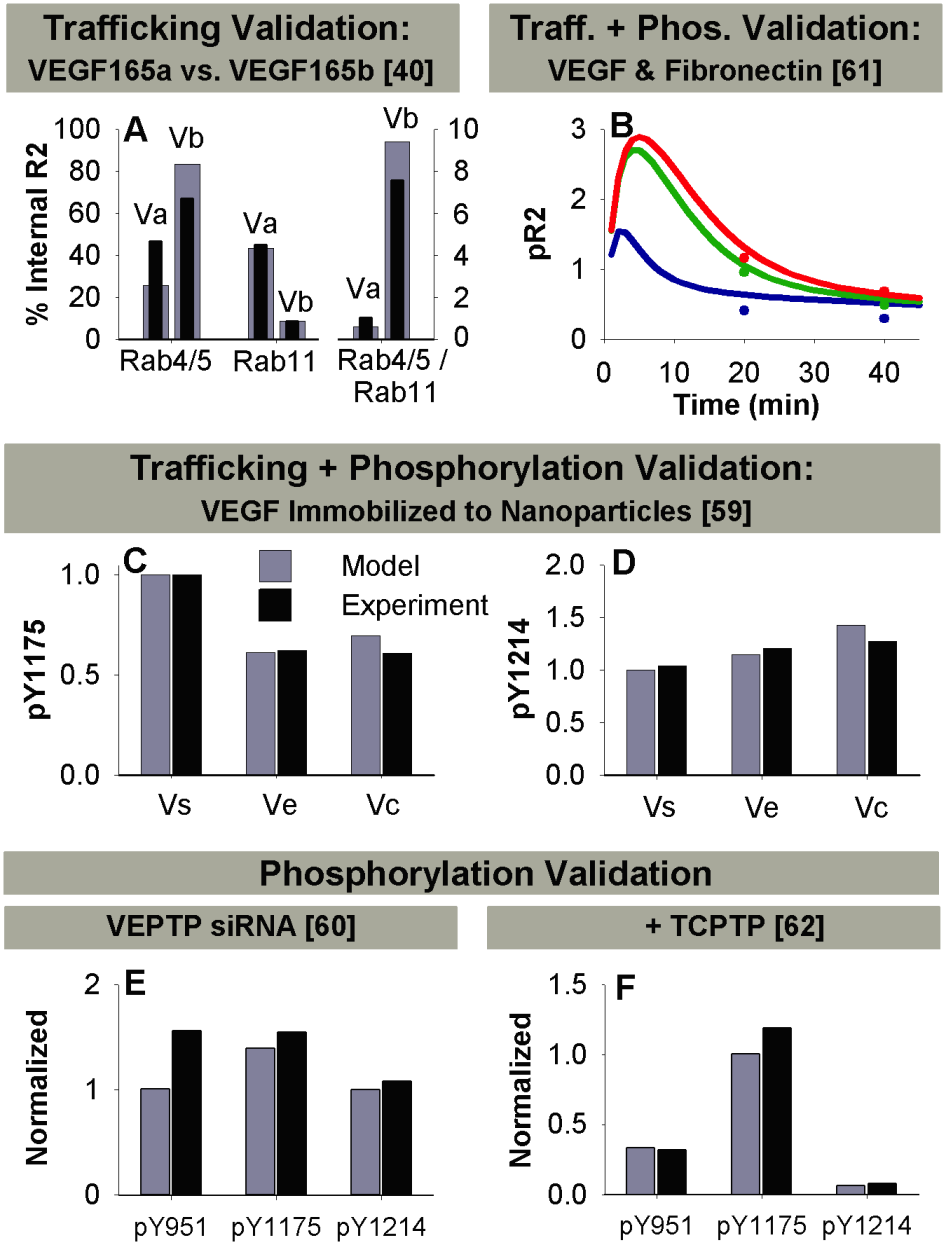
Validation of complete model with trafficking and phosphorylation parameters. A. Validation of trafficking parameters by comparing model predictions for VEGFR2 in the absence of NRP1 in PAECs compared to Ballmer-Hofer data for V_165b_ (V_b_), which does not bind NRP1. Data is compared to the normal case including NRP1 in the model and data for V_165a_ (V_a_), which does bind NRP1, at 30 min in the trafficking study [40]. The percent of internal VEGFR2 in Rab4/5 and Rab11 endosomes (left) and the ratio of total VEGFR2 in Rab4/5 endosomes to total VEGFR2 in Rab11 endosomes (right) is shown. Grey: model simulations; black, experimental data. B-D. Validation of complete model including trafficking and phosphorylation parameters. B) Data taken from Martino et. al. 2011 [61]. [V] = 50 ng/mL. Assumed VEGF and FN are pre-mixed. Soluble VEGF, blue; VEGF bound to wild type fibronectin, green; VEGF bound to rFNIII9-10/12-14 fragments, red (k_off,M·V_ values from Wijelath et. al. 2006 [18]). C-D. Data taken from an additional Anderson et. al. 2011 study [59]. E-F. Validation of phosphorylation parameters by comparing model predictions to data in cells with perturbations to specific phosphatases. E. Impact of siRNA against VEPTP on pY951, pY1175, and pY1214. Experimental data (black) taken from Mellberg et. al. 2006 [60]. TIME cells, [V] = 50 ng/mL, measurements taken at t = 5 min. In the model, the experimentally observed decrease in VEPTP expression to 20% of control values with VEPTP siRNA was simulated by decreasing the dephosphorylation rate for Y951 and Y1175 by a factor of 5 on the cell surface. HUVEC receptor numbers were used in model. F. Impact of exposing HEK 293 cells transfected with VEGFR2 to a constitutively active form of TCPTP on pR2, pY1175, and pY1214. Data taken from Mattila et. al. 2008 [62]. In the model, we simulated the constitutively active TCPTP by increasing the dephosphorylation rate for Y951 and Y1214 to match the rate for unligated R2 (30 s^-1^) in all compartments. HUVEC receptor numbers were used in model.

To validate our trafficking parameters, we used additional data from the trafficking study. Here, PAECS transfected with NRP1 and VEGFR2 were stimulated with the VEGF isoform V_165b_ [40], which does not bind NRP1 [70]. We used our model to predict whether the differences in VEGFR2 distribution upon stimulation with V_165a_ (V) and V_165b_ can be accounted for solely by the inability of V_165b_ to bind NRP1 (Fig 4A and 4B) by comparing to simulations lacking NRP1. The ratio of VEGFR2 in Rab4/5 endosomes to VEGFR2 in Rab11 endosomes matched data for V_165b_ well (Fig 4A). Thus, the model predicts that the impact of V_165b_ on VEGFR2 localization in early and recycling endosomes (compared to V_165a_) can be accounted for solely by its inability to bind NRP1, and suggesting that our model framework can capture differences in trafficking between VEGF isoforms. The model overestimates routing of VEGFR2 for degradation (compared to Rab7 populations in the experimental data) for V_165a_ and underestimates it for V_165b_ (Fig 4A). This may indicate some error in the relative values of our degradation rate constants for V·R2 and V·N1·R2, due to the limited and normalized data available to fit these rate constants, but it does not affect model predictions when NRP1 is present.

Next, we validated the complete model including biochemical reactions, trafficking processes, and phosphorylation reactions using two additional studies of soluble and immobilized VEGF (see Methods) [59, 61] (Fig 4B–4D). We also validated our phosphorylation parameters by comparing model predictions to experimental measurements of VEGFR2 phosphorylation after perturbation of the expression or activity of phosphatases that are known to regulate VEGFR2. As we did not explicitly include these phosphatases in our model, we altered the dephosphorylation rate constants specifically for the tyrosine residues on which the specified phosphatase is known to act, and in the subcellular locations where the phosphatase is typically located. We examined two phosphatases known to act on VEGFR2, but that are not yet completely understood. VEPTP, which is found on the plasma membrane, dephosphorylates Y951 and Y1175, but not Y1214 [60]. TCPTP, which is found both at the plasma membrane and in the cytosol, dephosphorylates Y1214 and likely also Y951, but not Y1175 [62]. Fig 4E shows model predictions and experimental data for change in pY951, pY1175, and pY1214 when TIME cells are treated with siRNA to VEPTP. Application of VEPTP siRNA was modeled as a decrease in the dephosphorylation rate constants for Y951 and Y1175 on the cell surface by a factor of 5, to match the experimental observation that VEPTP siRNA results in reduction of VEPTP expression to 20% of expression in the control. Fig 4F shows model predictions of VEGFR2 phosphorylation in HEK 293 cells exposed to a constitutively active form of TCPTP, which was modeled by increasing the dephosphorylation rate constants for Y951 and Y1214 to the values for unligated VEGFR2 (30 s^-1^) in all compartments. The model predicts that VEPTP siRNA only partially abrogates dephosphorylation of VEGFR2, while addition of constitutively active TCPTP results in strong dephosphorylation of Y1214. The consistency of model predictions and experimental data in these studies provides further validation for our model framework.

### Trafficking-Mediated Regulation of Site-Specific Phosphorylation Is Sufficient to Capture Experimental Trends in VEGFR2 Activation

**Fig 5:**
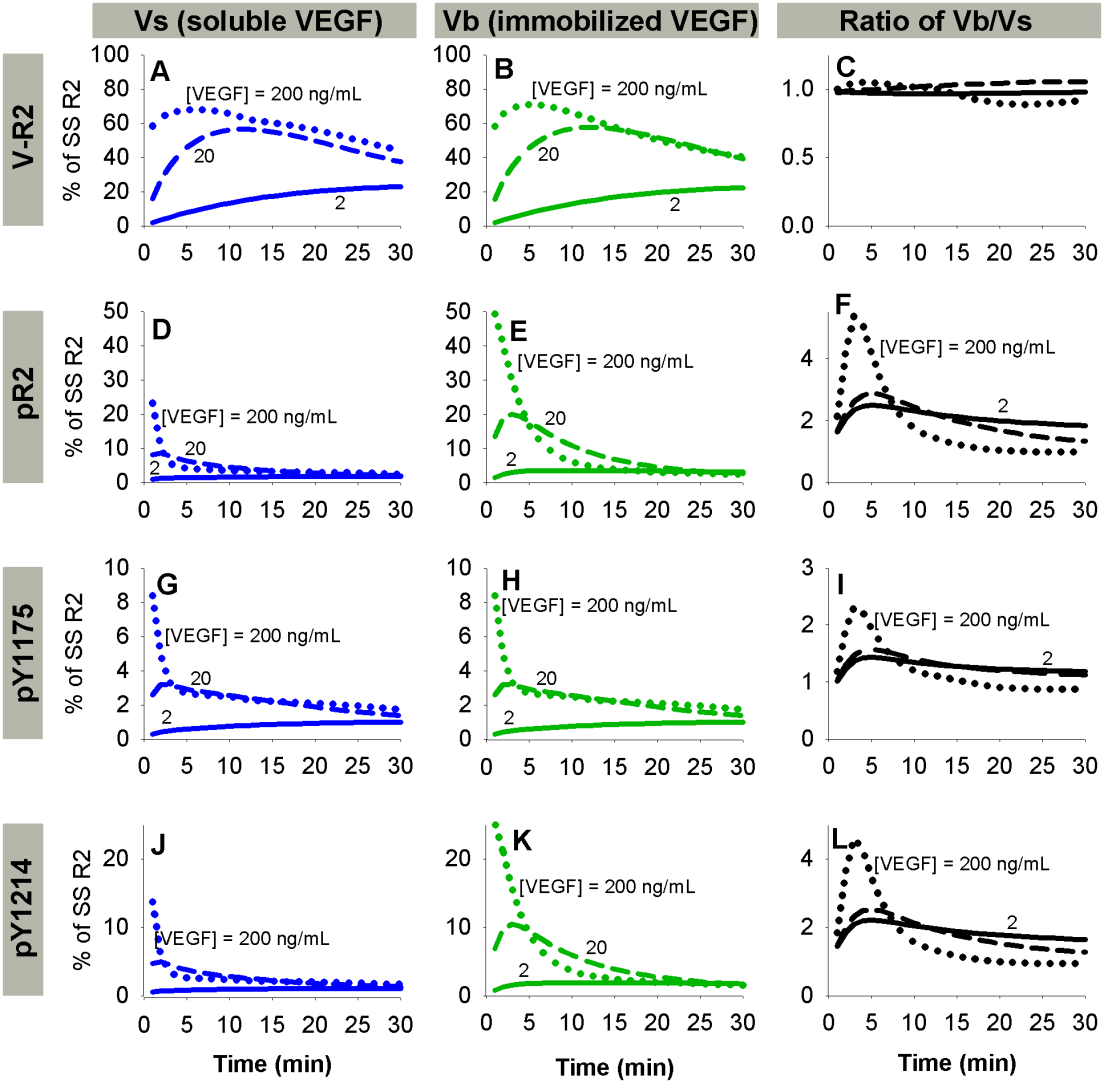
VEGF presentation mode affects VEGFR2 phosphorylation more than VEGFR2 ligation. The time-dependent response to soluble VEGF (Vs, blue, left column) and bound VEGF (Vb, green, middle column) of: VEGF-ligated VEGFR2 (V·R2, A-C); all phosphorylated VEGFR2 (pR2, D-F); and site-specific phosphorylated VEGFR2, pY1175 (G-I) and pY1214 (J-L). The ratios of responses to bound and soluble VEGF are shown at the right. Time-scale ends at 30 minutes, but pR2 curves are relatively flat after this time. Solid line, [V] = 2 ng/mL; dashed line, [V] = 20 ng/mL; dotted line, [V] = 200 ng/mL.

**Fig 6:**
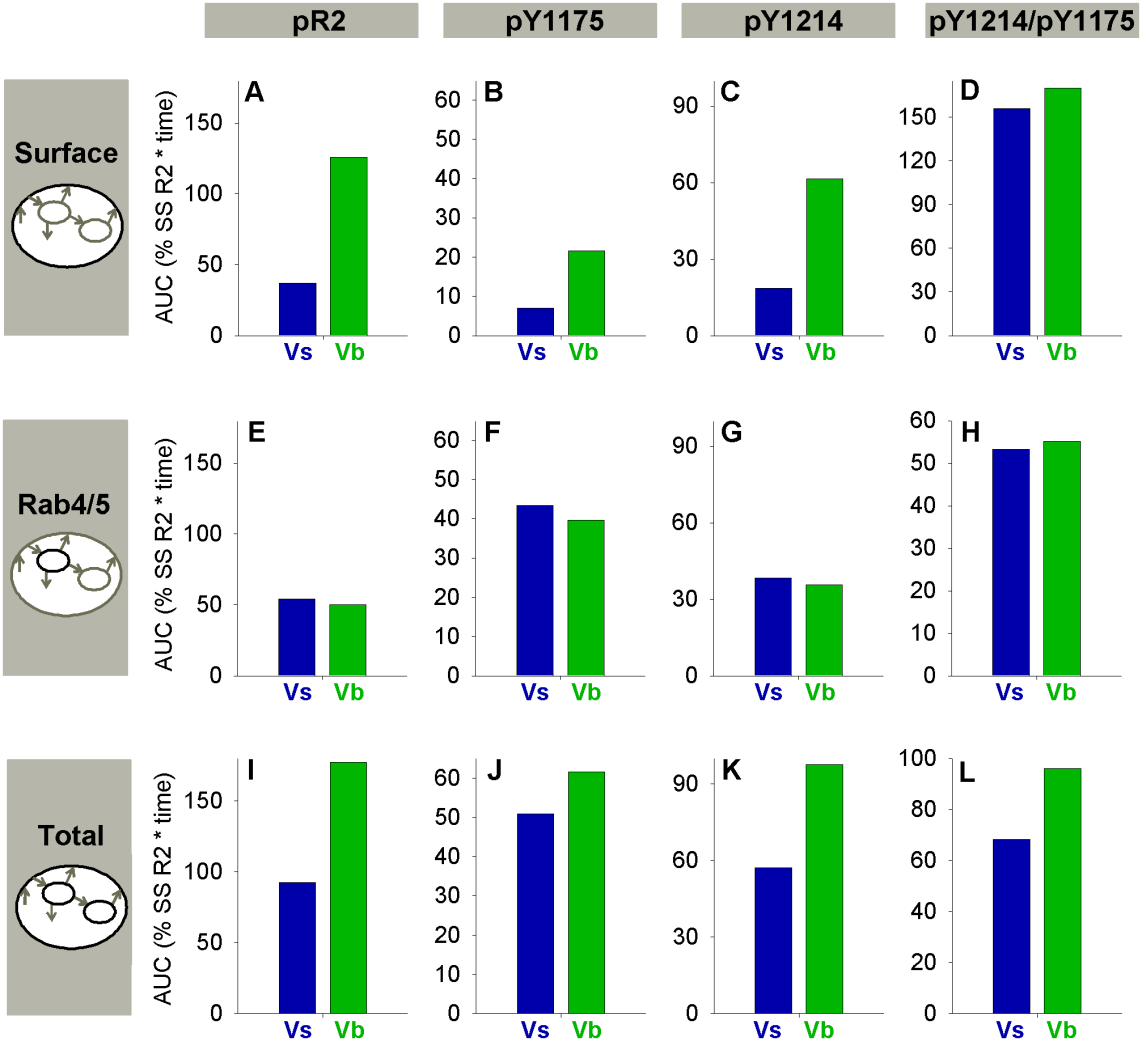
Increased total VEGFR2 activation with immobilized VEGF is driven by the change in surface VEGFR2. All panels show area under the curve (AUC), a measure of total VEGFR2 activation, for the first 60 minutes after stimulation with soluble (Vs- blue) or immobilized (Vb- green) VEGF at a concentration of 2 ng/mL. AUCs are shown for surface VEGFR2 (A-D), Rab4/5 VEGFR2 (E-H), and total VEGFR2 (I-L). Activation on any considered tyrosine residue (pR2, 1^st^ column), Y1175 (2^nd^ column), and Y1214 (3^rd^ column) are compared. The last column shows the AUC for the curve pY1214/pY1175 (total pY1214/pY1175 curves shown in Fig 8F and 8G) for surface VEGFR2 (D), Rab4/5 VEGFR2 (H), and total VEGFR2 (L). Note that the difference in total pY1214/pY1175 for Vs and Vb emerges from the altered VEGFR2 distribution, not altered pY1214/pY1175 in each subcellular location.

In order to understand the influence of trafficking on phosphorylation of VEGFR2, we compared the relative amounts of pR2, pY1175, and pY1214 in each subcellular location and in total after stimulation with soluble and immobilized VEGF (Fig 5, S3 Fig). While pY1175 and pY1214 are both split between the cell surface and Rab4/5 endosomes, Rab4/5 endosomes contain more pY1175 (S3H–S3I Fig, Fig 6B and 6C) and the cell surface has more pY1214 (S3E–S3F Fig, Fig 6F and 6G). The peak magnitude of total pY1214 (Fig 5K) is predicted to be more than twice the peak magnitude of pY1175 after stimulation with immobilized VEGF (Fig 5H, due to the increased surface population of VEGF·VEGFR2), but the peak magnitudes are more similar when stimulated with soluble VEGF (Fig 5G and 5J). Immobilization of VEGF also leads to increased pR2 peak magnitude on the cell surface (S3 Fig), decreased pR2 peak magnitude in Rab4/5 endosomes (S3 Fig), and increased duration of total pR2 (Fig 5D–5F, S3 Fig). To test the hypothesis that multiple endosomal compartments with distinct properties are necessary to capture experimentally observed trends in pY1175 and pY1214, we examined a case where the dephosphorylation rate constants in Rab11 endosomes were assumed to be the same as those in Rab4/5 endosomes (S4 Fig). In this case, peak pY1214 remains ssentially unchanged, but the duration increases (S4 Fig), while the peak magnitude and duration of pY1175 increases significantly (S4 Fig). The observed increase in duration is inconsistent with experimental data, emphasizing the need for multiple internal compartments with independent dephosphorylation rates to reflect the cellular localization of phosphatases.

The model clearly predicts that ligated VEGFR2 (V·R2) is not an accurate representation of VEGFR2 phosphorylated on at least one of the considered residues (pR2) at all times; while V·R2 and pR2 are both elevated at early times, the durations are quite dissimilar (Fig 5A–5F). We found that only 33% of ligated VEGFR2 is phosphorylated on at least of one Y951, Y1175, and Y1214 at 5 minutes after stimulation with 20 ng/mL of immobilized VEGF (S5 Fig). By 30 minutes, only 7% of ligated VEGFR2 is phosphorylated on one of these residues (S5B Fig), though a significant amount of VEGFR2 is predicted to remain ligated (Fig 2E–2M, Fig 5A and 5B). This model prediction is consistent with experimental observations that, while detectable pR2 is low by 30 minutes in most cases, total VEGF and VEGFR2 populations are still significant [29, 59]. To test the robustness of this prediction, we increased our assumed phosphorylation rate constant. This did not substantially increase the percentage of VEGF-VEGFR2 phosphorylated at 30 minutes (not shown). The short duration of phosphorylation on ligated VEGFR2 results from recycling through Rab11 endosomes, where dephosphorylation rates are high.

To quantify the total amount of VEGFR2 activation in the first 60 minutes after stimulation with soluble or immobilized VEGF, we calculated the area under the pR2 vs. time curves (curves shown in S3 Fig). The increase in total VEGFR2 activation observed after stimulation with Vb compared to Vs results primarily from increased pR2 on the surface (Fig 6A–6C, S6 Fig). Note that the relative proportion of total surface VEGFR2 activation on Y1175 and Y1214 is similar for Vs and Vb (Fig 6D); the difference in total signaling (Fig 6L) arises from the relative sizes of the surface and internal populations (Fig 2E–2M). The simulations also predicted that the size of the increases in the pY1214/pY1175 ratio due to immobilized VEGF is higher at lower VEGF concentrations (S6 Fig), which is where the physiological range of VEGF concentrations lies [13].

### Neuropilin-1 Modulates Site-Specific VEGFR2 Phosphorylation due to Altered Recycling and Degradation of VEGF-Bound VEGFR2

**Fig 7:**
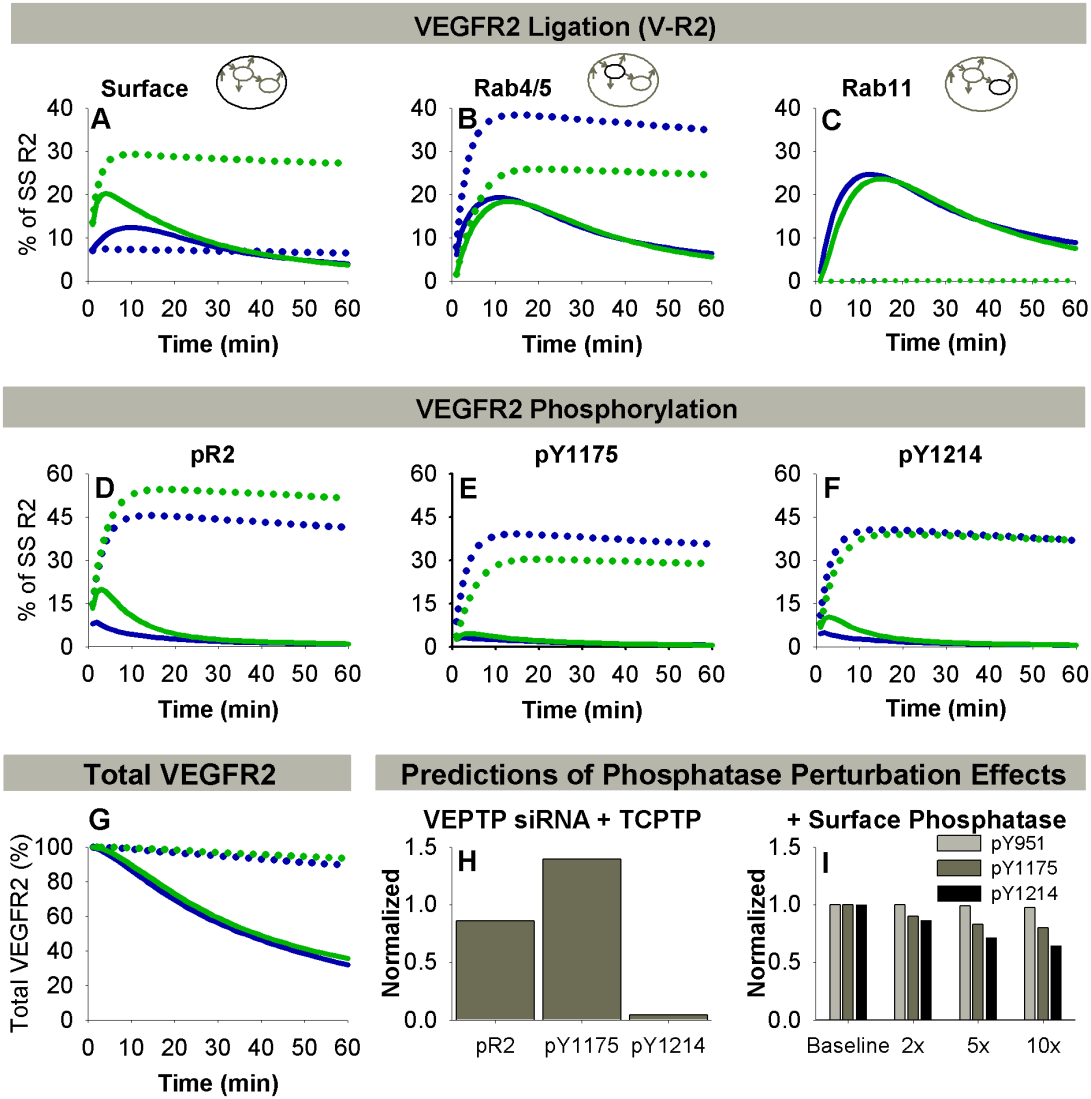
Neuropilin-1 and phosphatases modulate site-specific VEGFR2 phosphorylation. **A-C.** Distribution of VEGF-bound VEGFR2 (sum of V·R2, V·N1·R2, and M·V·R2) in HUVECs. Solid lines: Baseline case with NRP1 present; dotted lines: no NRP1 present. Soluble VEGF (Vs), blue lines; bound VEGF (Vb), green lines. For all lines, [V] = 20 ng/mL, HUVEC receptor numbers. **D-G.** Total VEGFR2 phosphorylated on at least one of Y951, Y1175, and Y1214 (pR2, D), pY1175 (E), pY1214 (F), and total VEGFR2 (G) with NRP1 (solid lines) and without NRP1 (dotted lines). **H-I.** Model predictions for site-specific VEGFR2 phosphorylation under perturbation of phosphatase activity. **H.** Model predictions for the experiment in Fig 4F (siRNA to VEPTP), with the addition of a constitutively active TCPTP (as described in Fig 4G). **I.** Model predictions for exposure of HUVECs to a cell-surface phosphatase that dephosphorylates Y951, Y1175, and Y1214, similar to DEP-1. The impact of increasing phosphatase expression by a factor of 2, 5, or 10 is shown.

In most endothelial cell types, NRP1 is present at cell surface densities higher than those of VEGFR2 [65]. Based on the strong receptor-receptor coupling effect, it is expected that the majority of cell surface V·R2 will be bound to NRP1 [12, 32]. To probe the impact of NRP1 on VEGFR2 trafficking and phosphorylation, we simulated stimulation of HUVECs with VEGF in the presence or absence of NRP1 (Fig 7A–7G). In the absence of NRP1 (e.g. through siRNA knockdown), the model predicts less total degradation of VEGFR2 (Fig 7G) and an increase in the duration of VEGFR2 phosphorylation (Fig 7D–7F, due to decreased degradation and decreased localization to Rab11 endosomes, where efficient dephosphorylation occurs). NRP1 absence also results in a larger difference in VEGFR2 ligation after stimulation with soluble or immobilized VEGF (Fig 7A–7C). In the absence of NRP1, V·R2 (Fig 7A–7C) and unligated VEGFR2 (S7 Fig) are predicted to accumulate in the Rab4/5 compartment (Fig 7B, S7 Fig), as the “fast” Rab11 recycling pathway is not accessible (k_4to11_ and k_rec11_ >> k_rec4_). Thus, our model supports the hypothesis that NRP1 is necessary for the enhancement of VEGFR2 recycling observed upon stimulation with VEGF (by rerouting the ligated receptor through the Rab11 recycling pathway) [40]. This is also consistent with experimental observations that Rab11-dependent recycling of VEGFR2 leads to increased activation of p38 after stimulation with soluble VEGF, presumably due to the return of more VEGFR2 to the plasma membrane and subsequent reactivation [40, 71]. Upon stimulation with immobilized VEGF in the absence of NRP1, surface VEGF-VEGFR2 is increased, as the matrix (M) does not have to compete with NRP1 for binding to VEGF (Fig 7A). These changes have the potential to alter the balance of downstream signaling (Fig 7E and 7F) differently than VEGF immobilization or NRP1 loss alone. By routing V·R2 through the Rab11 compartment to be dephosphorylated and recycled, NRP1 may be a strong regulator of pR2, and thus of interest as a therapeutic target.

As we assume that M and NRP1 cannot bind to VEGF simultaneously, we examined whether, for immobilized VEGF presentation, VEGF-bound VEGFR2 internalized following detachment from the matrix would be predicted to remain unbound to NRP1, potentially altering its recycling and degradation compared to soluble VEGF. The model predicts that almost all internal V·R2 complexes (Rab4/5 and Rab11) contain NRP1 after stimulation with either form of VEGF (S8 Fig). Thus, it is predicted that NRP1 joins the V·R2 complex shortly after dissociation of VEGF from M, so VEGF immobilization does not alter V·R2 recycling and degradation *after* dissociation from the matrix and internalization.

### Integrated Model Can Predict the Impact of Novel Phosphatase Perturbations on VEGFR2 Phosphorylation

We also used the model to predict the impact of novel scenarios involving phosphatases. First, we predicted the impact of exposing HUVECs to VEGF after treatment with a combination of siRNA to VEPTP and a constitutively active form of TCPTP (Fig 7H). The simulations show that pY1175 would be elevated, due to the VEPTP siRNA, while pY1214 would be decreased, due to the constitutively active TCPTP. This produces a situation where the balance of pY1175 and pY1214 is strongly shifted, while total phosphorylated VEGFR2 (pR2) remains close to baseline levels. We also explored the impact of a cell surface-localized phosphatase that dephosphorylated Y951, Y1175, and Y1214 (Fig 7I). This phosphatase is similar to DEP-1, except that DEP-1 is also implicated in inhibition of VEGFR2 internalization [46], which our theoretical phosphatase does not do. We examined the impact of this phosphatase by increasing the rate constant for cell surface dephosphorylation of all three residues in this model, in order to mimic increased expression of this phosphatase (Fig 7I). We found that pY1214 was decreased most by the phosphatase, while pY1175 was affected less because this phosphatase cannot access VEGFR2 in Rab4/5 endosomes. These model predictions are examples of specific mechanistic hypotheses that could be tested experimentally.

### VEGF Immobilization Alters Surface vs. Internal Distribution of VEGFR2 and Balance of pY1175 and pY1214

**Fig 8:**
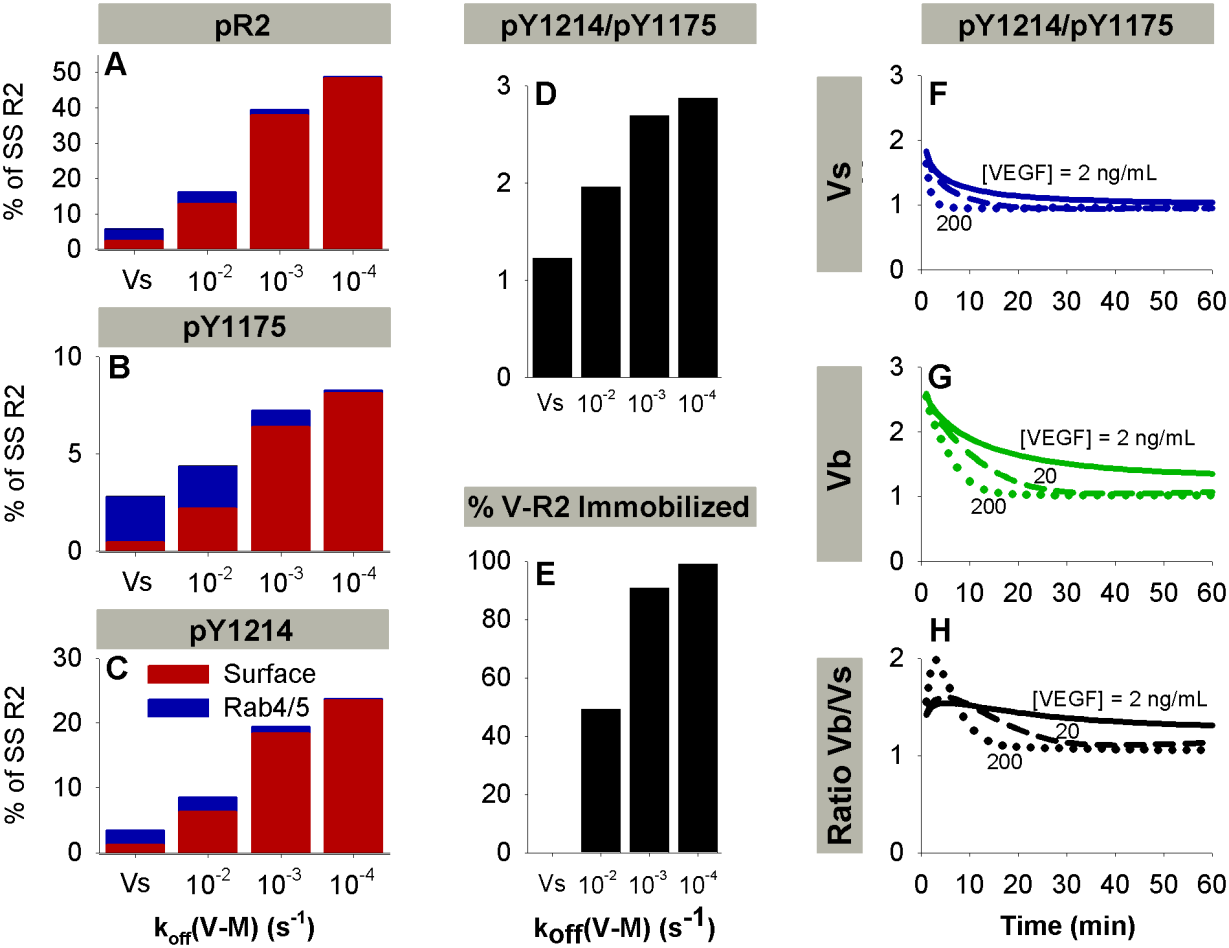
Relative activation pY1175 and pY1214 varies as a function of VEGF immobilization and concentration. **A-C.** Total VEGFR2 phosphorylated on at least one of Y951, Y1175, and Y1214 (pR2, A) and site-specific phosphorylation (pY1175, B and pY1214, C) at 5 min with varying k_off,V·M_. Note quantities of VEGFR2 phosphorylated on any residues in Rab11 endosomes are negligible. **D.** Ratio of pY1214 to pY1175 at 5 minutes with varying k_off,V·M_. **E.** Percent of surface V·R2 complexes that are bound to the matrix at t = 5 minutes. **F-H.** The ratio of pY1214-VEGFR2 to pY1175-VEGFR2 for Vs (F) and Vb (G). The ratio of this quantity for bound VEGF and soluble VEGF (Vb/Vs) is shown in (H). Solid line, [V] = 2 ng/mL; dashed line, [V] = 20 ng/mL; dotted line, [V] = 200 ng/mL. Soluble VEGF (Vs), blue lines; bound VEGF (Vb), green lines. For all lines, HUVEC receptor numbers were used.

There is significant interest in how specific immobilization techniques will alter activation of VEGFR2. As a first step, we examined the influence of varying the rate constant for VEGF release from the matrix, k_off,V·M_ (Fig 8A–8E). Assuming all immobilized VEGF is spatially available to bind VEGFR2, a significant portion of cell surface V·R2 is bound to the matrix (M) (Fig 8E). Total peak pY1175 magnitude and distribution across subcellular locations (Fig 8A and 8B), is significantly altered by increasing k_off,V·M_. pY1214 remains primarily surface-localized for all cases with immobilized VEGF (Fig 8C), but the magnitude decreases with increasing k_off,V·M_, altering the relative magnitudes of pY1175 and pY1214 (Fig 8D). While total ligated VEGFR2 is not dramatically different after stimulation with soluble or immobilized VEGF (Fig 5A–5C), the ratio pY1214/pY1175 is greater than two at early times after stimulation with immobilized VEGF, favoring p38 activation and migration (Fig 8G), while the ratio is closer to one for soluble VEGF, favoring ERK activation and proliferation (Fig 8F). Increasing k_off,V·M_ decreases the difference in pR2 after stimulation with soluble or immobilized VEGF (Fig 8A) by allowing for more internalization of VEGF·VEGFR2 complexes in the Vb case.

### pY1175 and pY1214 Are Predicted to Be More Sensitive to Trafficking Parameters Than to Phosphorylation Parameters

We performed a local sensitivity analysis to investigate which initial conditions and rate constants impact model outputs the most (SP Fig). The outputs considered were pY1175, pY1214, and the ratio pY1214/pY1175 after stimulation with soluble or immobilized VEGF at 5, 15, or 30 minutes. We found that sensitivities are notably different at low (2 ng/mL, S9 Fig) and high (200 ng/mL, S9 Fig) VEGF concentrations. Specifically, at low VEGF concentrations, outputs are more sensitive to initial conditions ([V] and receptor numbers) and to dephosphorylation parameters (S9 Fig); while at high VEGF concentrations, outputs are more sensitive to trafficking parameters (S9 Fig). Almost all outputs are quite sensitive to VEGFR2 and NRP1 levels. In general, model outputs (pY1175, pY1214, pY1214/pY1175) are less sensitive to the phosphorylation parameters than the trafficking parameters, reinforcing that, in our model, trafficking is a strong regulator of phosphorylation patterns. Stimulation with immobilized VEGF decreased the sensitivity of the model to trafficking parameters compared to soluble VEGF.

## Discussion

### Computational Model of VEGFR2 Ligation, Trafficking, and Phosphorylation

In this study we developed a computational model that quantitatively relates VEGF-VEGFR2 binding and trafficking of VEGFR2 to patterns of site-specific phosphorylation. Our model was parameterized using data from three separate studies [8, 29, 40], and validated against data from four additional independent studies [59–62]. We identified trafficking parameters that describe experimental data in multiple endothelial cell lines, suggesting that trafficking processes may be more conserved than previously thought. Our simulations predict that the relative surface and endosomal populations of ligated VEGFR2 (V·R2) are affected by the coreceptor NRP1. By routing V·R2 through Rab11 endosomes to be dephosphorylated and recycled, NRP1 may be a strong regulator of VEGFR2 phosphorylation. Our model further indicates that immobilization of VEGF leads to decreased internalization, but does not affect recycling or degradation of internalized receptors because ligated VEGFR2 (V·R2) is predicted to bind NRP1 quickly after unbinding from the matrix. The model predicts that the durations of ligated VEGFR2 (V·R2) and phosphorylated VEGFR2 (pR2) curves are quite dissimilar, providing a good rationale for simulating phosphorylation and dephosphorylation separately from receptor ligation. Additionally, the pR2 pool represents only a fraction of ligated VEGFR2 (<35% by 5 minutes after VEGF stimulation), and is altered by perturbations to dephosphorylation of specific residues.

In enabling us for the first time to predict VEGFR2 phosphorylation patterns in response to receptor ligation and trafficking, without assuming that phosphorylation of VEGFR2 is synonymous with ligation and dimerization, this study represents an advance over existing models. In our previous models of VEGF-NRP1-VEGFR2 interactions [12, 32, 72], we had not included the effect of NRP1 on VEGFR2 trafficking. The observed differences in duration of ligated (V·R2) and phosphorylated VEGFR2 (pR2) captured by our model cannot be replicated by a model with a single endosomal compartment. An early compartment where significant amounts of phosphorylated VEGFR2 can accumulate is necessary, while a second, later compartment is required where ligated VEGFR2 is dephosphorylated before recycling to the cell surface. This model is also the first to include site-specific activation of VEGFR2 by immobilized VEGF. Importantly, our model accurately captures *relative* trends in phosphorylation of Y1175 and Y1214 between soluble and immobilized VEGF (S2 Fig) at multiple VEGF concentrations (2–200 ng/mL) and under a variety of experimental conditions. A computational model developed by the authors of the 2011 presentation study captured trends in VEGF internalization between soluble and immobilized VEGF, but does not contain the level of detail in trafficking and phosphorylation included in our model, and cannot replicate trends in pY1175 and pY1214 (Fig 3B and 3C) [8]. Most of the phosphorylation data used to fit our model was normalized and semi-quantitative; as such, we cannot validate model predictions of the total percentage of VEGFR2 phosphorylated, but we can make and validate predictions about the relative phosphorylation of VEGFR2 on Y1175 and Y1214, and how these curves change under various conditions. The detail in our model allows us to demonstrate that trafficking (and immobilization), coreceptor expression, and phosphatase activity all affect the balance of VEGFR2 phosphorylation on tyrosines 1175 and 1214. This in turn alters the recruitment of signaling complexes, and regulates downstream signaling and resultant cellular behavior. As such, changes to any combination of these could have therapeutic value, and all need to be considered in an integrated fashion when designing tissue constructs to promote development of a functional vascular network.

### Does Trafficking Regulate Phosphorylation, or Does Phosphorylation Regulate Trafficking?

Our simulations show how one ligand, VEGF, can elicit different cellular responses simply via changes in its mode of presentation. Differences in trafficking resulting from immobilization of VEGF are sufficient to account for differences in activation of VEGFR2 by soluble and matrix-bound VEGF. It is not necessary that there be a different activating VEGFR2 conformational change following soluble versus immobilized ligation. The link between immobilized/soluble ligands, receptor trafficking, and site-specific receptor tyrosine phosphorylation could be due to: (a) control of site-specific phosphorylation of VEGFR2 by receptor trafficking (e.g. via localization of site-specific phosphatases); or (b) conformational changes in VEGFR2 in response to ligation by matrix-bound ligands that result in altered site-specific phosphorylation of VEGFR2 compared to soluble ligands, which in turn alters trafficking of the receptor; or a combination of these mechanisms. Because phosphorylation is a fast process compared to trafficking, in line with previous estimates for EGFR [66] (see kinetics in Tables 2 and 3), receptor location and site-specific phosphorylation are highly correlated. This makes distinguishing between mechanisms (a) and (b) difficult. There are experimental results to date that support both (a) [8, 29, 40, 43] and (b) [46, 64, 73, 74]. Our model simulation results show that mechanism (a) is sufficient to explain all current experimental phosphorylation and trafficking data after stimulation with VEGF_165a_. This does not exclude (b), but suggests that it is not necessary. Alteration of the conformation of VEGFR2 in response to ligation with immobilized VEGF would be difficult to prove or disprove with current experimental techniques. However, the observation that immobilized VEGF effectively activates VEGFR2 whether it is coupled to different matrix molecules or even directly to a surface strongly suggests that the immobilized ligand is not specifically altered in conformation and that the receptor is not binding to an epitope that includes both the ligand and the matrix/surface. This reduces the likelihood of a ‘matrix-specific’ altered VEGFR2 conformation. An additional possibility is that immobilization of VEGF may interfere sterically with VEGFR2 coupling to regulatory molecules (e.g. integrins, VE-Cadherin and DEP-1), which may alter the phosphorylation pattern and/or trafficking of VEGFR2. Although internalization is known to be phosphorylation-dependent [64], complex control of trafficking by site-specific phosphorylation seems unlikely because it would require active sorting of VEGFR2 into vesicles by multi-tyrosine site phosphorylation pattern and specific internalization mechanics for each phospho-form of the receptor. Mechanism (a), on the other hand, relies only on passive sorting because matrix-binding VEGF retains VEGFR2 at the surface.

We propose an experiment that could, with the aid of our computational model, distinguish between (a) and (b) for specific trafficking steps and tyrosine residues on VEGFR2. The subcellular distribution of wild type VEGFR2 and VEGFR2 tyrosine mutants (e.g. Y1214F) could be compared in a manner similar to that in the trafficking study [40]. Differences in the localization of wild type (WT) and mutant VEGFR2 would suggest that phosphorylation on the mutated residue is required for the trafficking step where a change is observed. Computational modeling could predict whether additional changes are occurring downstream of the primary observed trafficking change. It would be important to verify that these mutants have equivalent ligand-binding and NRP1-coupling behaviors as WT VEGFR2. This proposed experiment would help to determine in more detail which mechanisms control each step of trafficking and phosphorylation, and to further refine our model.

The role of NRP1 is a confounding factor; it has been shown that treatment with a tyrosine kinase inhibitor (TKI) reduces VEGFR2-NRP1 complex formation in the presence of VEGF_165_ [75], which would in turn affect trafficking (and possibly also phosphorylation directly). This could imply that phosphorylation of VEGFR2 is required for complex formation with NRP1, or that TKIs interfere with interactions between the cytoplasmic domains of VEGFR2 and NRP1. The NRP1 cytoplasmic domain is required for binding of synectin and myosin VI, which are required for movement into EEA1-positive early endosomes and Rab4-to-Rab11 transfer, and thus NRP1-mediated control of VEGFR2 trafficking [33]. It is also possible that NRP1-binding could affect the phosphorylation pattern of VEGFR2 directly, which could then induce the trafficking changes mediated by NRP1. This is supported by evidence that VEGF_165b_, which does not bind Neuropilin-1, results in differential site-specific phosphorylation of VEGFR2 [70]. We suggest that additional experiments should be performed to clarify whether NRP1 directly alters phosphorylation of VEGFR2, and whether specific tyrosine residues are necessary for VEGFR2-NRP1 coupling.

### Model Insights for In Vivo Therapeutic Applications

**Fig 9:**
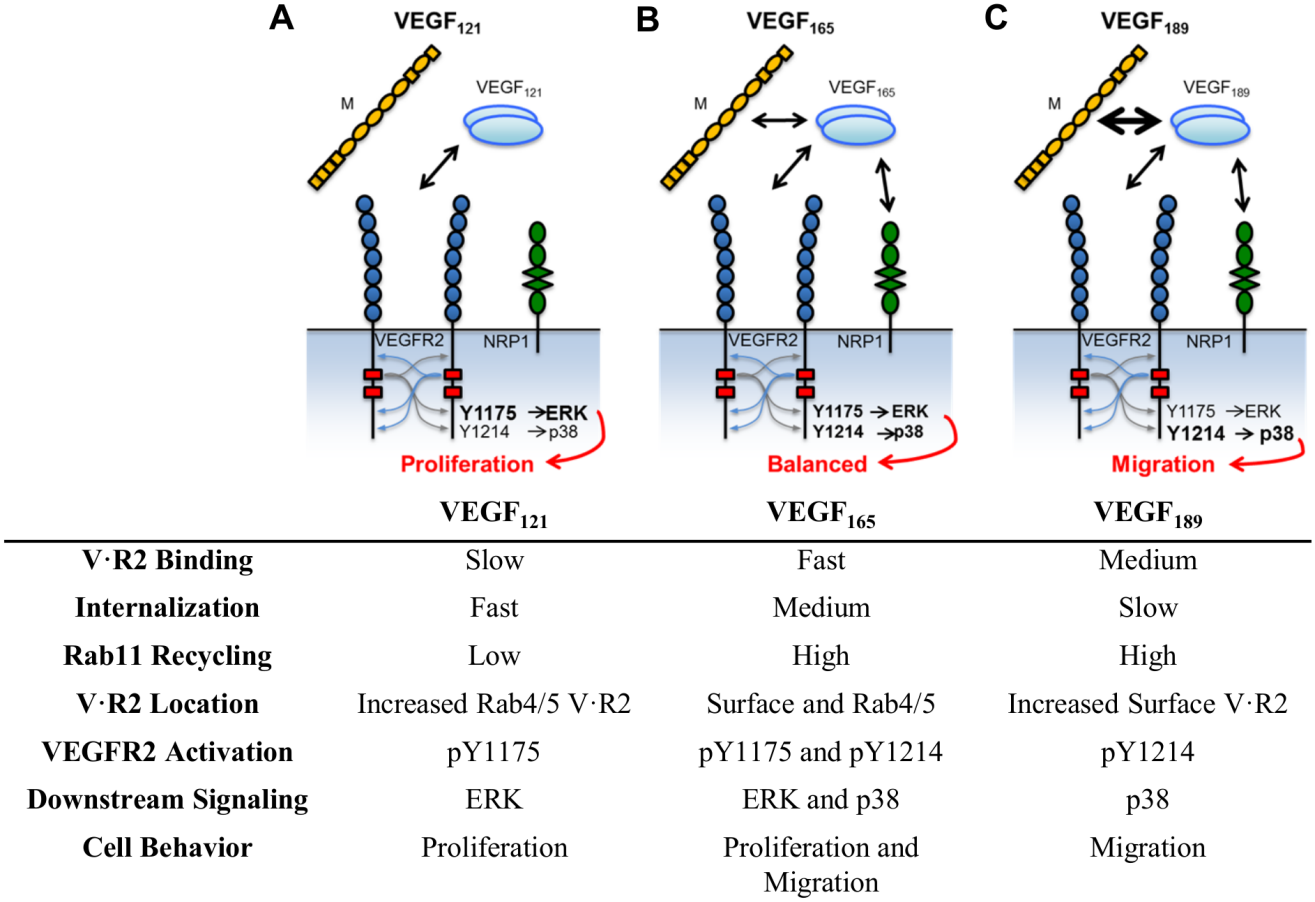
Differences in molecular interactions of VEGF isoforms are predicted to account for changes in observed vascular phenotype. **A.** VEGF_121_ does not bind NPR1 or extracellular matrix proteins (M), leading to slow VEGFR2 ligation (due to lack of NRP1-mediated VEGF-VEGFR2 binding), fast internalization (no immobilization of VEGF_121_), and a lack of recycling via the Rab11 pathway. **B.** VEGF_165_ binds both NRP1 and extracellular matrix species, leading to faster VEGFR2 ligation, but slower internalization of VEGFR2 compared to stimulation with VEGF_121_. The Rab11 recycling pathway is accessible to VEGF_165_-NRP1-VEGFR2 complexes. **C.** VEGF_189_ binds to extracellular matrix species and NRP1 more strongly than VEGF_165_. This results in moderate VEGFR2 ligation speed (NRP1 must compete with matrix species (M) for VEGF) and slow VEGFR2 internalization.

The majority of VEGF in normal tissues is sequestered in the ECM, so elucidating how immobilization alters site-specific phosphorylation of VEGFR2 is key to understanding VEGF behavior in tissues [12]. While the *in vitro* experimental studies examined here used equivalent concentrations of soluble and immobilized VEGF, the differences in concentrations and spatial availability of soluble and immobilized VEGF *in vivo* may result in a different balance of signaling in the body. VEGF_165_ is the most highly expressed isoform in normal tissue (though a significant portion of this may be VEGF_165b_ [67]), but VEGF_188/189_, which binds even more strongly to ECM species, represents a significant portion of the local VEGF in the mouse lung, heart, and liver, and in certain human tumors [20, 21]. The results of this study suggest that many of the differences in cell behavior and vascular morphology resulting from stimulation with different splice isoforms of VEGF may be direct effects of differences in interactions with Neuropilin-1 and the extracellular matrix (Fig 9). VEGF_121_ (Fig 9A) does not bind to NRP1 and VEGFR2 simultaneously, or to the matrix (M), resulting in slower ligand-receptor binding and faster internalization. We predict VEGF_121_-ligated VEGFR2 will localize to Rab4/5 endosomes, leading to increased Y1175 phosphorylation, ERK activation, and proliferation. The result would be a network of vessels with large diameters and little branching, as is seen in V_121_ isoform-specific mice [22, 23]. VEGF_165_ (Fig 9B), examined in this study, binds to both NRP1 and the matrix, resulting in fast VEGFR2 ligation and slower internalization compared to VEGF_121_. VEGF_165_ leads to a more even balance of surface and Rab4/5-located VEGF·VEGFR2, depending upon the strength of VEGF immobilization. This leads to a balance of pY1175 and pY1214, mixed ERK and p38 signaling, and a mix of cell proliferation and migration. VEGF_189_ (Fig 9C) binds strongly to the matrix and to NRP1. Competition between NRP1 and the matrix for VEGF slows VEGFR2 ligation and internalization. This increases pY1214 relative to pY1175, increasing p38 signaling and cell migration, and creating a highly branched vascular network, as is observed in VEGF_189_-specific mice [21, 22].

VEGF has potential utility in tissue engineering applications, where vascularization is necessary for the viability of thick tissue constructs. The ability to spatially organize the impetus to endothelial cells to proliferate and migrate could be used to effectively induce hierarchical networks with diverse diameters and branching properties. Current platforms for VEGF immobilization include tunable heparin-functionalized gold surfaces [8], collagen and PLGA hydrogels functionalized with VEGF [8, 29, 59], fibrin gels with active and passive growth factor release mechanisms, and presentation of VEGF with fibronectin fragments, VEGF binding peptides, and ECM proteins [11, 61, 76]. Cell lines, VEGF concentrations, quantities measured, time-points, and immobilization techniques vary across these studies. In aggregate, these experiments have demonstrated that immobilized VEGF promotes increased and extended phosphorylation of VEGFR2 [18, 29, 61, 77], specifically on Y1214 [8, 29, 59], activation of p38 [29, 59] and ERK [16, 18, 61, 78], and increased migration and proliferation [16, 18, 61, 77]. Results are less consistent regarding the impact of VEGF immobilization on phosphorylation of VEGFR2 on Y1175 [8, 29, 59] and activation of Akt [29, 78]. These conflicting results, combined with the limited success in producing functional vascular networks to date, make it clear that a better understanding of how VEGF immobilization alters cellular response to VEGF is essential to design effective scaffolds for regenerative applications requiring vascularization. In addition to reducing internalization, VEGF immobilization alters VEGFR2 cell surface interactions with co-receptors (NRP1, integrins, etc.). We begin to describe the impact of NRP1 on VEGFR2 trafficking, but more work remains to develop a quantitative understanding of VEGFR2-integrin interactions [79, 80], and how these interactions are altered by immobilization of VEGF. In the future, we can also examine the impact of ephrin B2 [81], epsins [82], dynamin2 [83], synectin and myosin VI [84], and NRP1 presentation *in trans* [85] on VEGFR2 activation by simulating their effects on VEGFR2 trafficking and phosphorylation.

Tyrosine phosphatases represent a pool of potential therapeutic targets that are not yet well-understood [49]. Targeting phosphatases to selectively control dephosphorylation of specific VEGFR2 tyrosine residues is appealing for anti-angiogenic therapies, which are currently focused mostly on antagonists to VEGF or to the receptor tyrosine kinases, but also for the control of therapeutic vascularization. Our model accurately captures the impact of perturbations to TCPTP and VEPTP on site-specific VEGFR2 phosphorylation (Fig 4E and 4F). While many other phosphatases are implicated in regulation of VEGFR2 phosphorylation (S5 Table), these two examples support our model’s structure of site- and location-specific dephosphorylation, and its ability to make therapeutically-relevant predictions.

Using this experimentally-validated model, we identified multiple key levers that can be manipulated to produce the desired multi-pathway signaling profile in endothelial and other cells. We identify VEGF presentation, trafficking, co-receptors (including NRP1), and regulatory molecules (such as phosphatases) as important levers that together control site-specific phosphorylation of VEGFR2 in a predictable way. While all of these facets are interconnected, we create a framework to study how perturbations to one or more of these levers alters VEGFR2 signaling. Future work is needed to tie changes in site-specific phosphorylation of Y951, Y1175, and Y1214 to activation of downstream signaling molecules. This work will continue to move us towards a more complete view of the VEGF system, improving our ability to design and predict the outcomes of novel vascular therapies.

## Supporting Information

S1 Fig Free and ligated VEGFR2 are not uniformly distributed between cell compartments. These panels expand on the results shown in Fig 2 of the main manuscript. Plots show the distribution of VEGF- bound VEGFR2 (V·R2, top row) and unbound VEGFR2 (Free R2, bottom row) on the cell surface (A,D), in Rab4/5 endosomes (B,E), and in Rab11 endosomes (C,F). In each case, the Y axis depicts the percentage of the VEGFR2 distributed to each compartment relative to the total VEGFR2 present at steady state (100%). After VEGF stimulation, percentages add up to less than 100, as a portion of the VEGFR2 has been degraded. Note that while peak levels of VEGFR2 ligation increase monotonically with VEGF concentration (solid line, [V] = 2 ng/mL; dashed line, [V] = 20 ng/mL; dotted line, [V] = 200 ng/mL), the temporal response is delayed at lower concentrations. Soluble VEGF (Vs), blue lines; bound VEGF (Vb), green lines.
(PDF)

S2 Fig Distribution of phosphorylation parameters. These panels expand on the results shown in Fig 3 of the main manuscript. **A.** Dephosphorylation parameters fit to experimental data. 47 sets of parameters were accepted as achieving good fit. Red dots indicate a representative coherent set of parameters used throughout the rest of this study. See Methods. **B.** Ratios of surface-to-internal dephosphorylation rates (panel A) for Y951, Y1175, and Y1214. The ratio of these ratios for Y1175 and Y1214 is also shown. The surface-to-internal dephosphorylation ratio is consistently higher for Y1175 than for Y1214.
(PDF)

S3 Fig Altered trafficking of VEGFR2 regulates site-specific phosphorylation of VEGFR2. These panels expand on the results show in Fig 5 of the main manuscript. Distribution of VEGFR2 phosphorylated on at least one of Y951, Y1175, and Y1214 (pR2, left), pY1175 (middle), or pY1214 (right) in total (top row), on the cell surface only (2^nd^ row), in Rab4/5 endosomes (3^rd^ row), and in Rab11 endosomes (4^th^ row) in HUVECs. Inset figures are included where the larger scale prevents clear distinction between lines. Time-scale ends at 30 minutes, but pR2 curves are relatively flat after this time. Soluble VEGF (Vs), blue line; bound VEGF (Vb), green line. Solid line, [V] = 2 ng/mL; dashed line, [V] = 20 ng/mL; dotted line, [V] = 200 ng/mL.
(PDF)

S4 Fig Independent receptor dephosphorylation rates in multiple internal compartments result in decreased pY1175-VEGFR2. These panels expand on the results shown in Fig 5 of the main manuscript. Impact on total phosphorylated VEGFR2 (pR2, A), pY1175-VEGFR2 (B), and pY1214-VEGFR2 (C) if k_dp_ in Rab11 endosomes is the same as in Rab4/5 endosomes. Solid Lines: Baseline case with dephosphorylation rates in each compartment as specified in Table 3 of the main manuscript; dotted lines: dephosphorylation rates in Rab11 endosomes set to the same values as for Rab4/5 endosomes. Soluble VEGF (Vs), blue lines; bound VEGF (Vb), green lines. For all lines, [V] = 20 ng/mL, HUVEC receptor numbers.
(PDF)

S5 Fig Only a fraction of ligated VEGFR2 is phosphorylated. These panels expand on the results shown in Fig 5 of the main manuscript. Percentage of ligated VEGFR2 is that is phosphorylated on at least one tyrosine residue for soluble VEGF (Vs, A) and matrix-bound VEGF (Vb, B). Quantities are broken down into cell surface, Rab4/5 endosome, and Rab11 endosome components. Quantities of phosphorylated VEGFR2 are negligible in Rab11 endosomes. For all lines, [V] = 20 ng/mL, HUVEC receptor numbers.
(PDF)

S6 Fig Trends in ligated and phosphorylated VEGFR2 are consistent across VEGF concentrations. These panels expand on the results show in Fig 6 of the main manuscript. All panels show area under the curve (AUC), for the first 60 minutes after stimulation with soluble (Vs- blue) or immobilized (Vb- green) VEGF at concentrations of 2, 20, and 200 ng/mL. AUCs are shown for cell surface quantities (A-F), Rab4/5 quantities (G-L), and total quantities (M-R). AUCs are shown for total VEGFR2 (1^st^ column), ligated VEGFR2 (2^nd^ column), VEGFR2 phosphorylation on any considered tyrosine residue (pR2, 3^rd^ column), pY1175 (4^th^ column), and pY1214 (5^th^ column). The last column shows the AUC for the curve pY1214/pY1175 (not the ratio AUC for pY1175 / AUC for pY1214) for surface VEGFR2 (F), Rab4/5 VEGFR2 (L), and total VEGFR2 (R).
(PDF)

S7 Fig Loss of NRP1 increases levels of free VEGFR2 on the cell surface and in Rab4/5 endosomes. These panels expand on the results shown in Fig 7 of the main manuscript. Distribution of unligated VEGFR2. Solid lines: Baseline case with NRP1 present; dotted lines: no NRP1 present. Soluble VEGF (Vs), blue lines; bound VEGF (Vb), green lines. For all lines, [V] = 20 ng/mL, HUVEC receptor numbers.
(PDF)

S8 Fig The majority of ligated VEGFR2 is complexed with NRP1. These panels expand on the results shown in Fig 7 of the main manuscript. Percentages of VEGFR2 that are ligated and immobilized (M·V·R2, green), bound to NRP1 (V·N1·R2, blue), or bound to neither M nor NRP1 (V·R2, red), upon stimulation with soluble VEGF (Vs, left) or immobilized VEGF (Vb, right) at 5 minutes (top) and 15 minutes (bottom). Values shown are percentages of the total steady-state VEGFR2 population in the specified compartment and at the given times. Note that even upon stimulation with matrix-bound VEGF, essentially all internal ligated VEGFR2 is complexed with NRP1, due to the excess of NRP1. [V] = 20 ng/mL, HUVEC receptor numbers.
(PDF)

S9 Fig Sensitivity of model outputs varies with VEGF concentration. Sensitivity of outputs to small changes in parameters and initial conditions. Values are average fold change in selected outputs for a 1-fold change in the specified parameter. **A.** [V] = 2 ng/mL. Scale: Black = 0, Bright red (maximum) = 0.77 **B.** [V] = 200 ng/mL. Scale: Black = 0, Bright red = 1.03 (maximum).
(PDF)

S1 Table Molecules included in the model and simulations. (DOCX)

S2 Table Cell geometry parameters. (DOCX)

S3 Table Representative fits to experimental trafficking data. (DOCX)

S4 Table Summary of distribution of accepted parameter sets. (DOCX)

S5 Table Summary of phosphatases acting on VEGFR2. (DOCX)

S1 Equations (PDF)
