# Supplementary material for: Site-Specific Phosphorylation of VEGFR2 Is Mediated by Receptor Trafficking: Insights from a Computational Model
Source: PLoS Comput Biol. 2015 Jun 12;11(6):e1004158. doi: 10.1371/journal.pcbi.1004158 (PMC4466579; doi:10.1371/journal.pcbi.1004158)
Supplement: S1 Fig — These panels expand on the results shown in Fig 2 of the main manuscript. Plots show the distribution of VEGF- bound VEGFR2 (V·R2, top row) and unbound VEGFR2 (Free R2, bottom row) on the cell surface (A,D), in Rab4/5 endosomes (B,E), and in Rab11 endosomes (C,F). In each case, the Y axis depicts the percentage of the VEGFR2 distributed to each compartment relative to the total VEGFR2 present at steady state (100%). After VEGF stimulation, percentages add up to less than 100, as a portion of the VEGFR2 has been degraded. Note that while peak levels of VEGFR2 ligation increase monotonically with VEGF concentration (solid line, [V] = 2 ng/mL; dashed line, [V] = 20 ng/mL; dotted line, [V] = 200 ng/mL), the temporal response is delayed at lower concentrations. Soluble VEGF (Vs), blue lines; bound VEGF (Vb), green lines. (PDF) [file pcbi.1004158.s001.pdf]

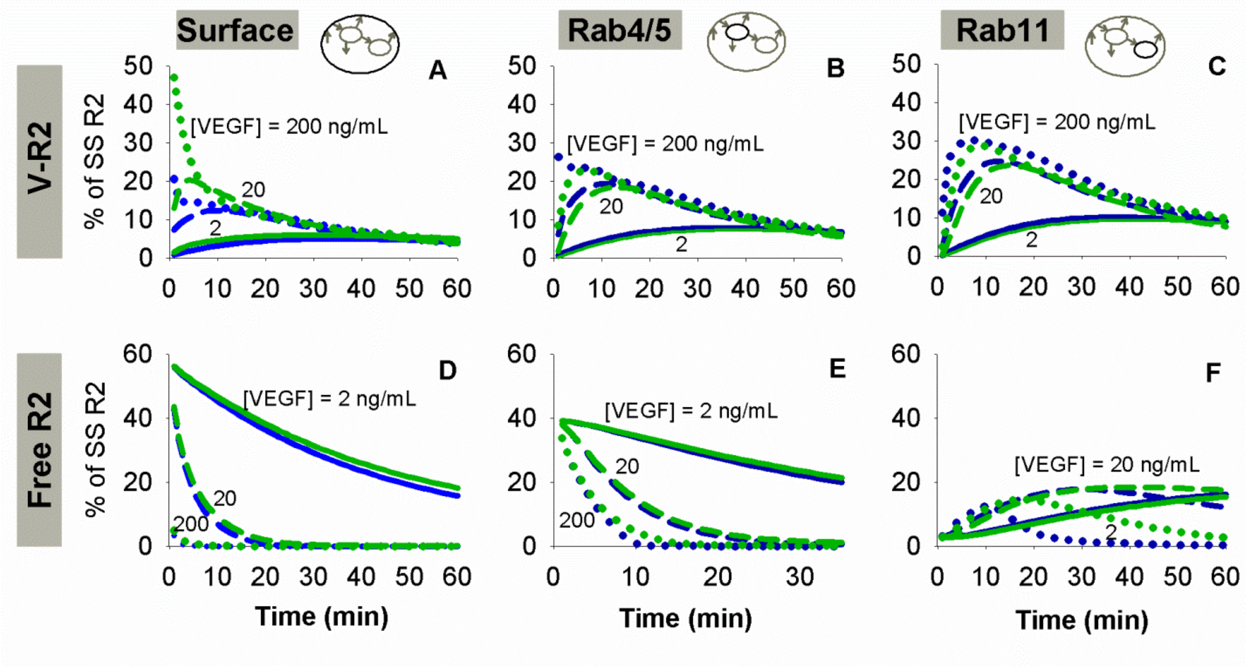

Figure S1. Free and ligated VEGFR2 are not uniformly distributed between cell compartments. These panels expand on the results shown in Figure 2 of the main manuscript. Plots show the distribution of VEGF- bound VEGFR2 (V·R2, top row) and unbound VEGFR2 (Free R2, bottom row) on the cell surface (A,D), in Rab4/5 endosomes (B,E), and in Rab11 endosomes (C,F). In each case, the Y axis depicts the percentage of the VEGFR2 distributed to each compartment relative to the total VEGFR2 present at steady state (100%). After VEGF stimulation, percentages add up to less than 100, as a portion of the VEGFR2 has been degraded. Note that while peak levels of VEGFR2 ligation increase monotonically with VEGF concentration (solid line, [V] = 2 ng/mL; dashed line, [V] = 20 ng/mL; dotted line, [V] = 200 ng/mL), the temporal response is delayed at lower concentrations. Soluble VEGF (Vs), blue lines; bound VEGF (Vb), green lines.
