# Supplementary material for: Site-Specific Phosphorylation of VEGFR2 Is Mediated by Receptor Trafficking: Insights from a Computational Model
Source: PLoS Comput Biol. 2015 Jun 12;11(6):e1004158. doi: 10.1371/journal.pcbi.1004158 (PMC4466579; doi:10.1371/journal.pcbi.1004158)
Supplement: S2 Fig — These panels expand on the results shown in Fig 3 of the main manuscript. A. Dephosphorylation parameters fit to experimental data. 47 sets of parameters were accepted as achieving good fit. Red dots indicate a representative coherent set of parameters used throughout the rest of this study. See Methods. B. Ratios of surface-to-internal dephosphorylation rates (panel A) for Y951, Y1175, and Y1214. The ratio of these ratios for Y1175 and Y1214 is also shown. The surface-to-internal dephosphorylation ratio is consistently higher for Y1175 than for Y1214. (PDF) [file pcbi.1004158.s002.pdf]

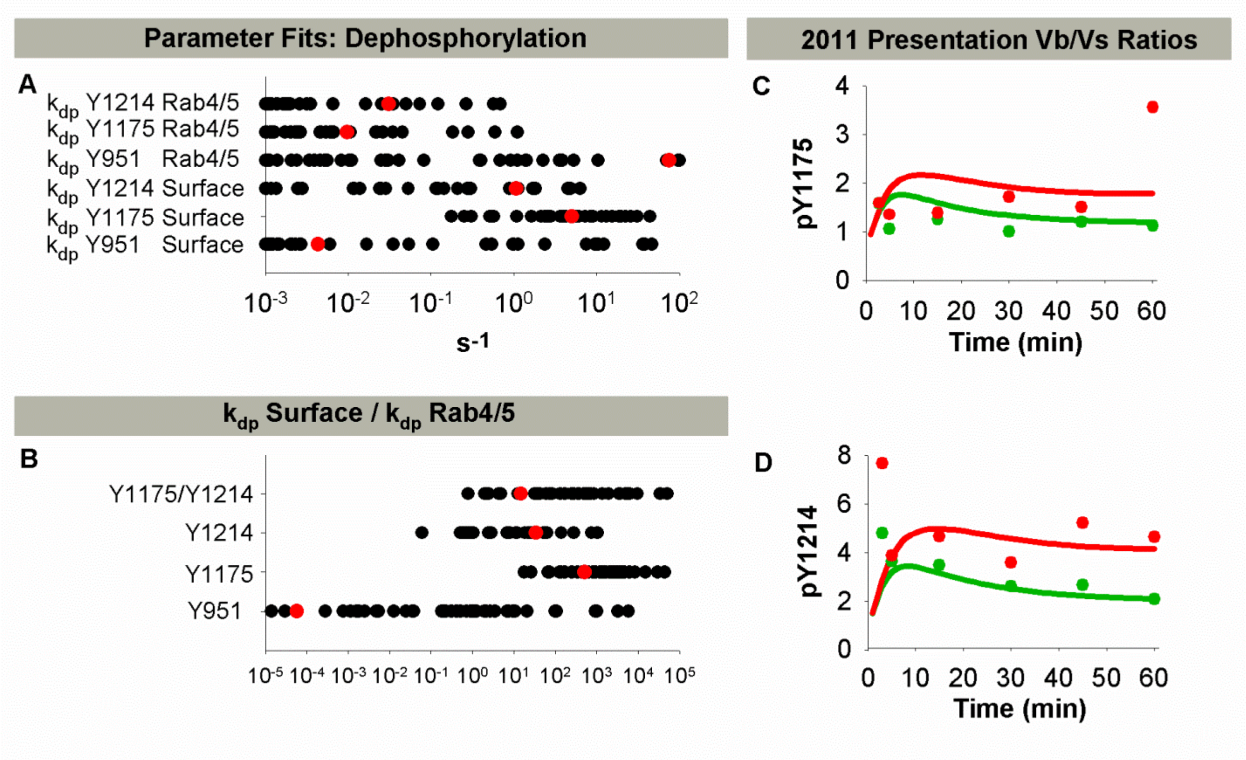

Figure S2. Distribution of Phosphorylation Parameters. These panels expand on the results shown in Figure 3 of the main manuscript. **A.** Dephosphorylation parameters fit to experimental data. 47 sets of parameters were accepted as achieving good fit. Red dots indicate a representative coherent set of parameters used throughout the rest of this study. *See* Methods. **B.** Ratios of surface-to-internal dephosphorylation rates (panel A) for Y951, Y1175, and Y1214. The ratio of these ratios for Y1175 and Y1214 is also shown. The surface-to-internal dephosphorylation ratio is consistently higher for Y1175 than for Y1214. **C-D.** Ratios of immobilized VEGF (Vb) / soluble VEGF (Vs) for pY1175 (C) and pY1214 (D) from the 2011 presentation study [8]. Green, electrostatically immobilized VEGF; red, covalently immobilized VEGF. Lines, simulation; dots, experimental data. These ratios, along with the data in Fig. 3B-C, H were used to fit the dephosphorylation parameters.
