# Supplementary material for: Site-Specific Phosphorylation of VEGFR2 Is Mediated by Receptor Trafficking: Insights from a Computational Model
Source: PLoS Comput Biol. 2015 Jun 12;11(6):e1004158. doi: 10.1371/journal.pcbi.1004158 (PMC4466579; doi:10.1371/journal.pcbi.1004158)
Supplement: S3 Fig — These panels expand on the results show in Fig 5 of the main manuscript. Distribution of VEGFR2 phosphorylated on at least one of Y951, Y1175, and Y1214 (pR2, left), pY1175 (middle), or pY1214 (right) in total (top row), on the cell surface only (2nd row), in Rab4/5 endosomes (3rd row), and in Rab11 endosomes (4th row) in HUVECs. Inset figures are included where the larger scale prevents clear distinction between lines. Time-scale ends at 30 minutes, but pR2 curves are relatively flat after this time. Soluble VEGF (Vs), blue line; bound VEGF (Vb), green line. Solid line, [V] = 2 ng/mL; dashed line, [V] = 20 ng/mL; dotted line, [V] = 200 ng/mL. (PDF) [file pcbi.1004158.s003.pdf]

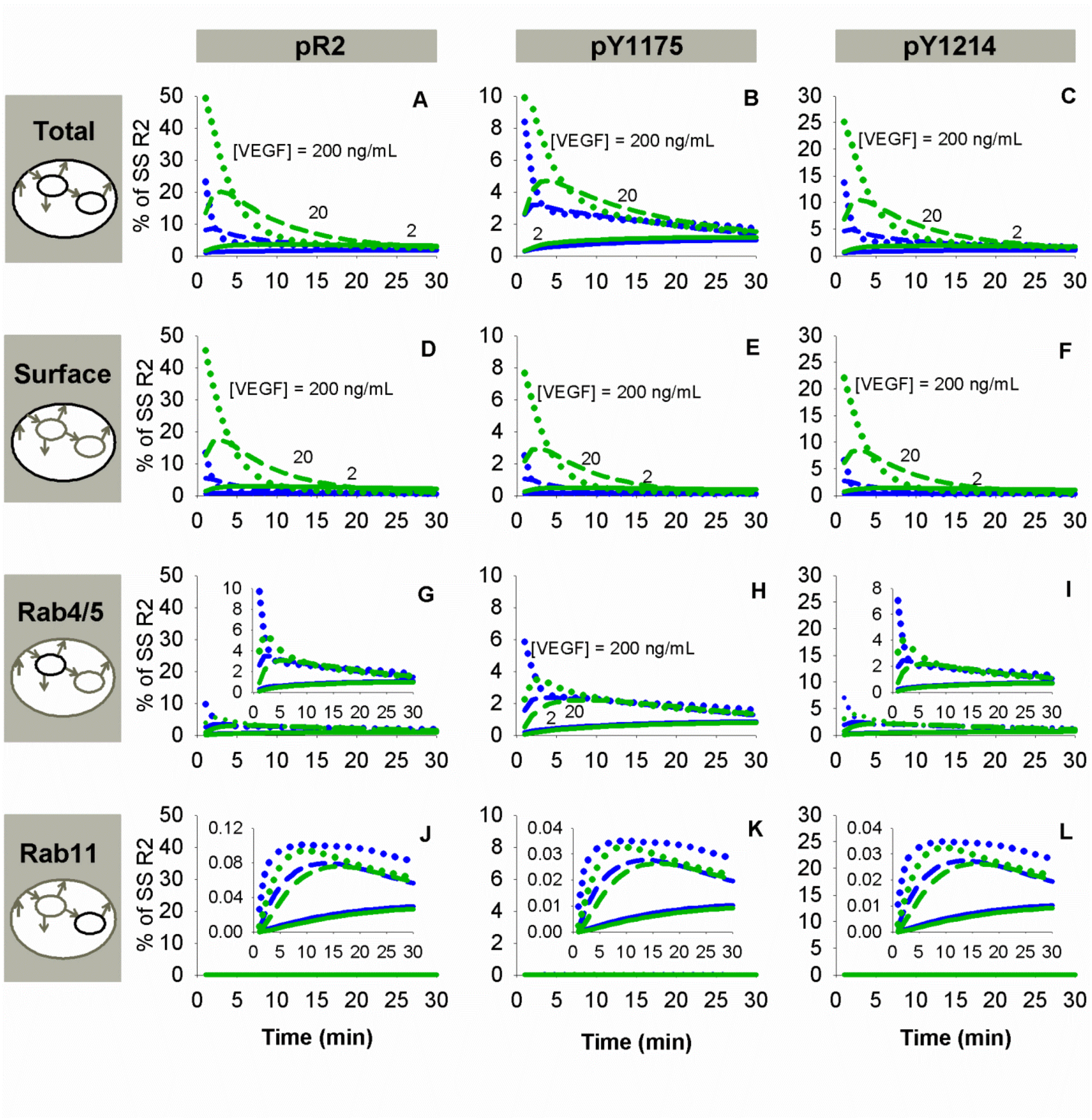

Figure S3. Altered trafficking of VEGFR2 regulates site-specific phosphorylation of VEGFR2. These panels expand on the results show in Figure 5 of the main manuscript. Distribution of VEGFR2 phosphorylated on at least one of Y951, Y1175, and Y1214 (pR2, left), pY1175 (middle), or pY1214 (right) in total (top row), on the cell surface only (2nd row), in Rab4/5 endosomes (3rd row), and in Rab11 endosomes (4th row) in HUVECs. Inset figures are included where the larger scale prevents clear distinction between lines. Time-scale ends at 30 minutes, but pR2 curves are relatively flat after this time. Soluble VEGF (Vs), blue line; bound VEGF (Vb), green line. Solid line, [V] = 2 ng/mL; dashed line, [V] = 20 ng/mL; dotted line, [V] = 200 ng/mL.
