# Supplementary material for: Site-Specific Phosphorylation of VEGFR2 Is Mediated by Receptor Trafficking: Insights from a Computational Model
Source: PLoS Comput Biol. 2015 Jun 12;11(6):e1004158. doi: 10.1371/journal.pcbi.1004158 (PMC4466579; doi:10.1371/journal.pcbi.1004158)
Supplement: S4 Fig — These panels expand on the results shown in Fig 5 of the main manuscript. Impact on total phosphorylated VEGFR2 (pR2, A), pY1175-VEGFR2 (B), and pY1214-VEGFR2 (C) if kdp in Rab11 endosomes is the same as in Rab4/5 endosomes. Solid Lines: Baseline case with dephosphorylation rates in each compartment as specified in Table 3 of the main manuscript; dotted lines: dephosphorylation rates in Rab11 endosomes set to the same values as for Rab4/5 endosomes. Soluble VEGF (Vs), blue lines; bound VEGF (Vb), green lines. For all lines, [V] = 20 ng/mL, HUVEC receptor numbers. (PDF) [file pcbi.1004158.s004.pdf]

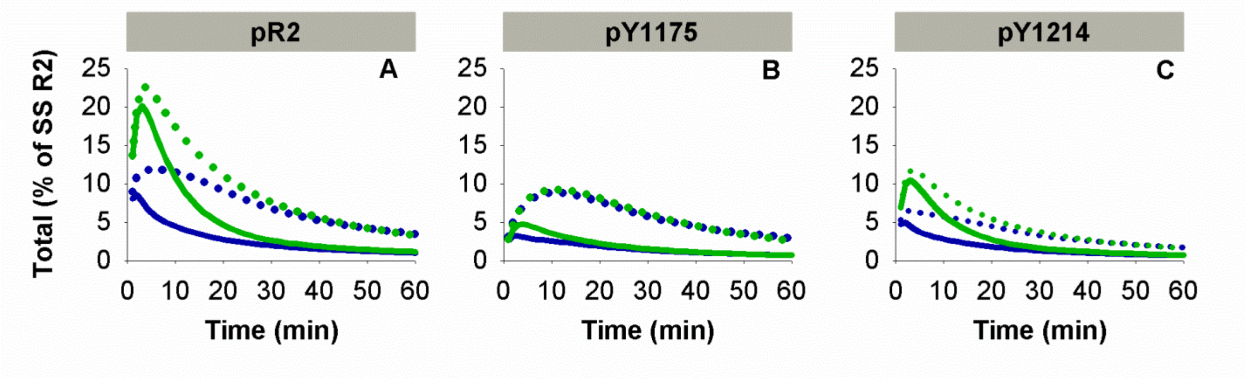

Figure S4. Independent receptor dephosphorylation rates in multiple internal compartments result in decreased pY1175-VEGFR2. These panels expand on the results shown in Figure 5 of the main manuscript. Impact on total phosphorylated VEGFR2 (pR2, A), pY1175-VEGFR2 (B), and pY1214-VEGFR2 (C) if k_{dp} in Rab11 endosomes is the same as in Rab4/5 endosomes. Solid Lines: Baseline case with dephosphorylation rates in each compartment as specified in Table 3 of the main manuscript; dotted lines: dephosphorylation rates in Rab11 endosomes set to the same values as for Rab4/5 endosomes. Soluble VEGF (Vs), blue lines; bound VEGF (Vb), green lines. For all lines, [V] = 20 ng/mL, HUVEC receptor numbers.
