# Supplementary material for: Site-Specific Phosphorylation of VEGFR2 Is Mediated by Receptor Trafficking: Insights from a Computational Model
Source: PLoS Comput Biol. 2015 Jun 12;11(6):e1004158. doi: 10.1371/journal.pcbi.1004158 (PMC4466579; doi:10.1371/journal.pcbi.1004158)
Supplement: S5 Fig — These panels expand on the results shown in Fig 5 of the main manuscript. Percentage of ligated VEGFR2 is that is phosphorylated on at least one tyrosine residue for soluble VEGF (Vs, A) and matrix-bound VEGF (Vb, B). Quantities are broken down into cell surface, Rab4/5 endosome, and Rab11 endosome components. Quantities of phosphorylated VEGFR2 are negligible in Rab11 endosomes. For all lines, [V] = 20 ng/mL, HUVEC receptor numbers. (PDF) [file pcbi.1004158.s005.pdf]

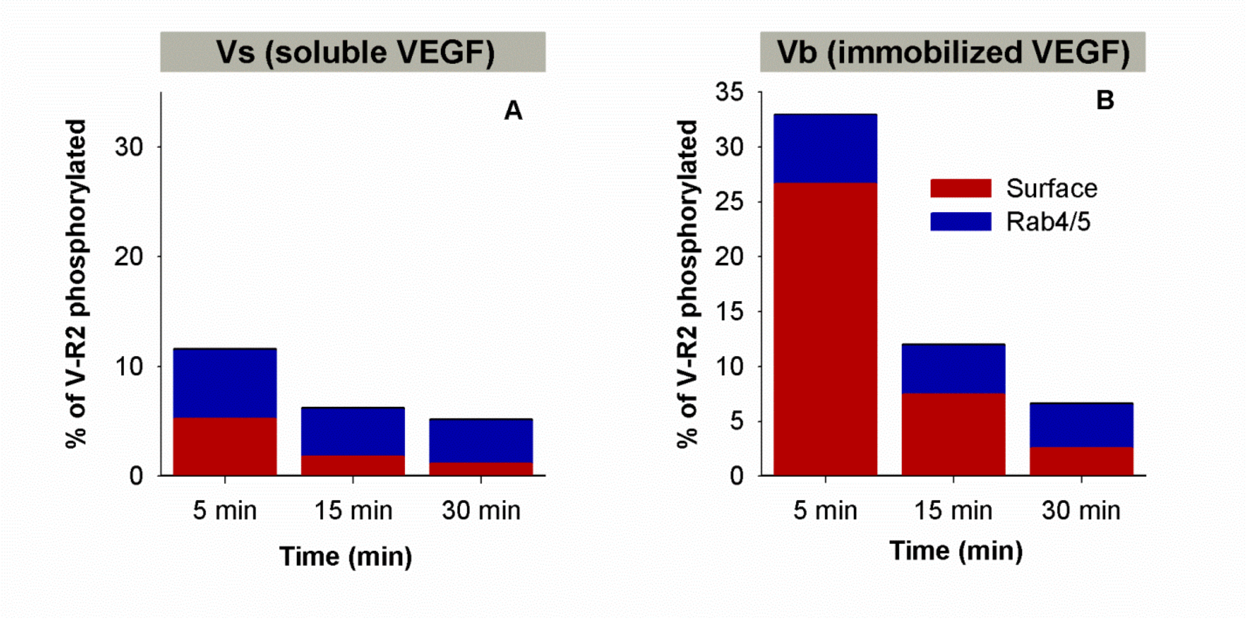

Figure S5. Only a fraction of ligated VEGFR2 is phosphorylated. These panels expand on the results shown in Figure 5 of the main manuscript. Percentage of ligated VEGFR2 is that is phosphorylated on at least one tyrosine residue for soluble VEGF (Vs, A) and matrix-bound VEGF (Vb, B). Quantities are broken down into cell surface, Rab4/5 endosome, and Rab11 endosome components. Quantities of phosphorylated VEGFR2 are negligible in Rab11 endosomes. For all lines, [V] = 20 ng/mL, HUVEC receptor numbers.
