# Supplementary material for: Site-Specific Phosphorylation of VEGFR2 Is Mediated by Receptor Trafficking: Insights from a Computational Model
Source: PLoS Comput Biol. 2015 Jun 12;11(6):e1004158. doi: 10.1371/journal.pcbi.1004158 (PMC4466579; doi:10.1371/journal.pcbi.1004158)
Supplement: S6 Fig — These panels expand on the results show in Fig 6 of the main manuscript. All panels show area under the curve (AUC), for the first 60 minutes after stimulation with soluble (Vs- blue) or immobilized (Vb- green) VEGF at concentrations of 2, 20, and 200 ng/mL. AUCs are shown for cell surface quantities (A-F), Rab4/5 quantities (G-L), and total quantities (M-R). AUCs are shown for total VEGFR2 (1st column), ligated VEGFR2 (2nd column), VEGFR2 phosphorylation on any considered tyrosine residue (pR2, 3rd column), pY1175 (4th column), and pY1214 (5th column). The last column shows the AUC for the curve pY1214/pY1175 (not the ratio AUC for pY1175 / AUC for pY1214) for surface VEGFR2 (F), Rab4/5 VEGFR2 (L), and total VEGFR2 (R). (PDF) [file pcbi.1004158.s006.pdf]

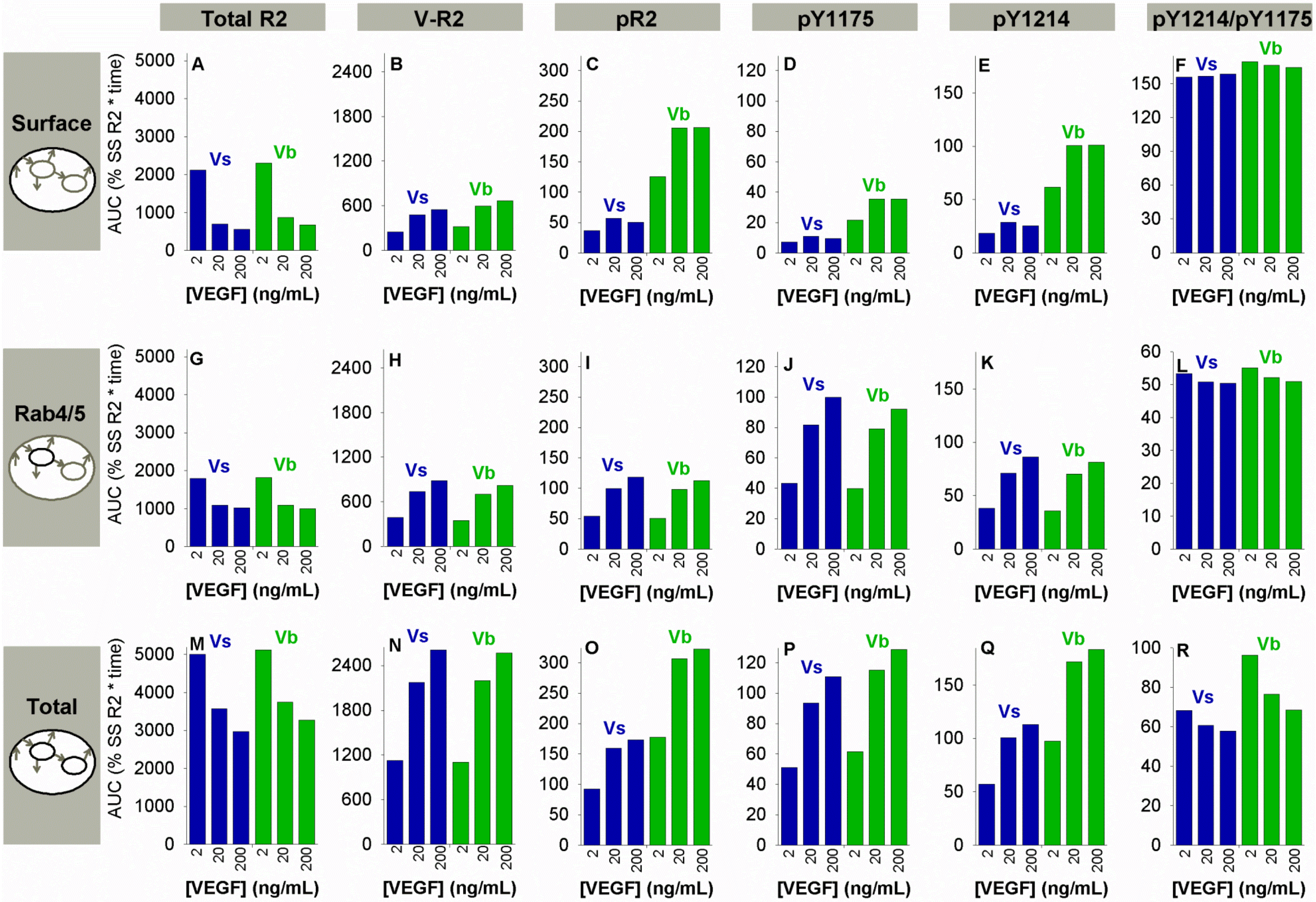

Total R2
V-R2
pR2
pY1175
pY1214
pY1214/pY1175
Surface
Rab4/5
Total
AUC (% SS R2 * time)
[VEGF] (ng/mL)
Vs
Vb
A
B
C
D
E
F
G
H
I
J
K
L
M
N
O
P
Q
R

Figure S6. Trends in ligated and phosphorylated VEGFR2 are consistent across VEGF concentrations. These panels expand on the results show in Figure 6 of the main manuscript. All panels show area under the curve (AUC), for the first 60 minutes after stimulation with soluble (Vs, blue) or immobilized (Vb, green) VEGF at concentrations of 2, 20, or 200 ng/mL. AUCs are shown for cell surface quantities (A-F), Rab4/5 quantities (G-L), and total quantities (M-R). AUCs are shown for total VEGFR2 (1st column), ligated VEGFR2 (2nd column), VEGFR2 phosphorylation on any considered tyrosine residue (pR2, 3rd column), pY1175 (4th column), and pY1214 (5th column). The last column shows the AUC for the curve pY1214/pY1175 (not the ratio AUC for pY1175 / AUC for pY1214) for surface VEGFR2 (F), Rab4/5 VEGFR2 (L), and total VEGFR2 (R).
