# Supplementary material for: Site-Specific Phosphorylation of VEGFR2 Is Mediated by Receptor Trafficking: Insights from a Computational Model
Source: PLoS Comput Biol. 2015 Jun 12;11(6):e1004158. doi: 10.1371/journal.pcbi.1004158 (PMC4466579; doi:10.1371/journal.pcbi.1004158)
Supplement: S7 Fig — These panels expand on the results shown in Fig 7 of the main manuscript. Distribution of unligated VEGFR2. Solid lines: Baseline case with NRP1 present; dotted lines: no NRP1 present. Soluble VEGF (Vs), blue lines; bound VEGF (Vb), green lines. For all lines, [V] = 20 ng/mL, HUVEC receptor numbers. (PDF) [file pcbi.1004158.s007.pdf]

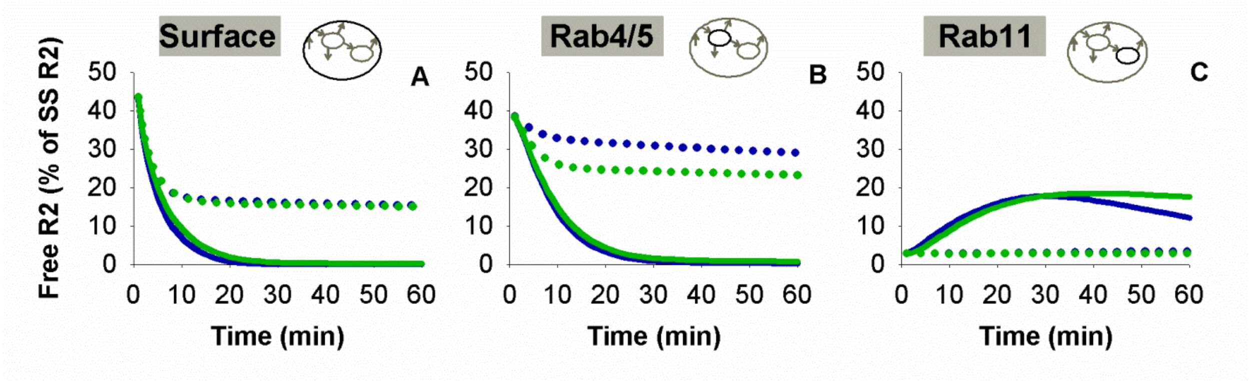

Figure S7. Loss of NRP1 increases levels of free VEGFR2 on the cell surface and in Rab4/5 endosomes. These panels expand on the results shown in Figure 7 of the main manuscript. Distribution of unligated VEGFR2. Solid lines: Baseline case with NRP1 present; dotted lines: no NRP1 present. Soluble VEGF (Vs), blue lines; bound VEGF (Vb), green lines. For all lines, [V] = 20 ng/mL, HUVEC receptor numbers.
