# Supplementary material for: Site-Specific Phosphorylation of VEGFR2 Is Mediated by Receptor Trafficking: Insights from a Computational Model
Source: PLoS Comput Biol. 2015 Jun 12;11(6):e1004158. doi: 10.1371/journal.pcbi.1004158 (PMC4466579; doi:10.1371/journal.pcbi.1004158)
Supplement: S8 Fig — These panels expand on the results shown in Fig 7 of the main manuscript. Percentages of VEGFR2 that are ligated and immobilized (M·V·R2, green), bound to NRP1 (V·N1·R2, blue), or bound to neither M nor NRP1 (V·R2, red), upon stimulation with soluble VEGF (Vs, left) or immobilized VEGF (Vb, right) at 5 minutes (top) and 15 minutes (bottom). Values shown are percentages of the total steady-state VEGFR2 population in the specified compartment and at the given times. Note that even upon stimulation with matrix-bound VEGF, essentially all internal ligated VEGFR2 is complexed with NRP1, due to the excess of NRP1. [V] = 20 ng/mL, HUVEC receptor numbers. (PDF) [file pcbi.1004158.s008.pdf]

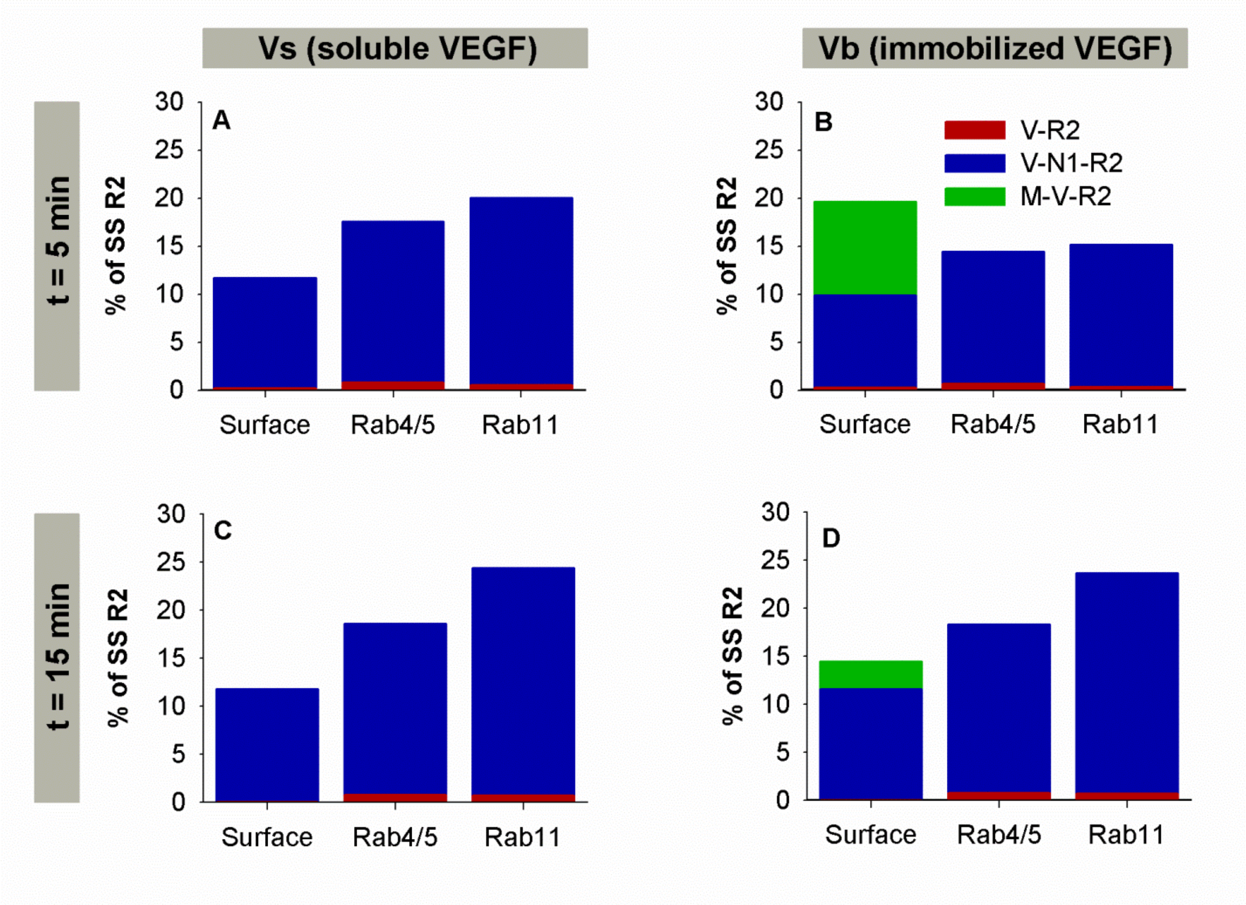

Figure S8. The majority of ligated VEGFR2 is complexed with NRP1. These panels expand on the results shown in Figure 7 of the main manuscript. Percentages of VEGFR2 that are ligated and immobilized (M·V·R2, green), bound to NRP1 (V·N1·R2, blue), or bound to neither M nor NRP1 (V·R2, red), upon stimulation with soluble VEGF (Vs, left) or immobilized VEGF (Vb, right) at 5 minutes (top) and 15 minutes (bottom). Values shown are percentages of the total steady-state VEGFR2 population in the specified compartment and at the given times. Note that even upon stimulation with matrix-bound VEGF, essentially all internal ligated VEGFR2 is complexed with NRP1, due to the excess of NRP1. [V] = 20 ng/mL, HUVEC receptor numbers.
