# Supplementary material for: Site-Specific Phosphorylation of VEGFR2 Is Mediated by Receptor Trafficking: Insights from a Computational Model
Source: PLoS Comput Biol. 2015 Jun 12;11(6):e1004158. doi: 10.1371/journal.pcbi.1004158 (PMC4466579; doi:10.1371/journal.pcbi.1004158)
Supplement: S9 Fig — Sensitivity of outputs to small changes in parameters and initial conditions. Values are average fold change in selected outputs for a 1-fold change in the specified parameter. A. [V] = 2 ng/mL. Scale: Black = 0, Bright red (maximum) = 0.77 B. [V] = 200 ng/mL. Scale: Black = 0, Bright red = 1.03 (maximum). (PDF) [file pcbi.1004158.s009.pdf]

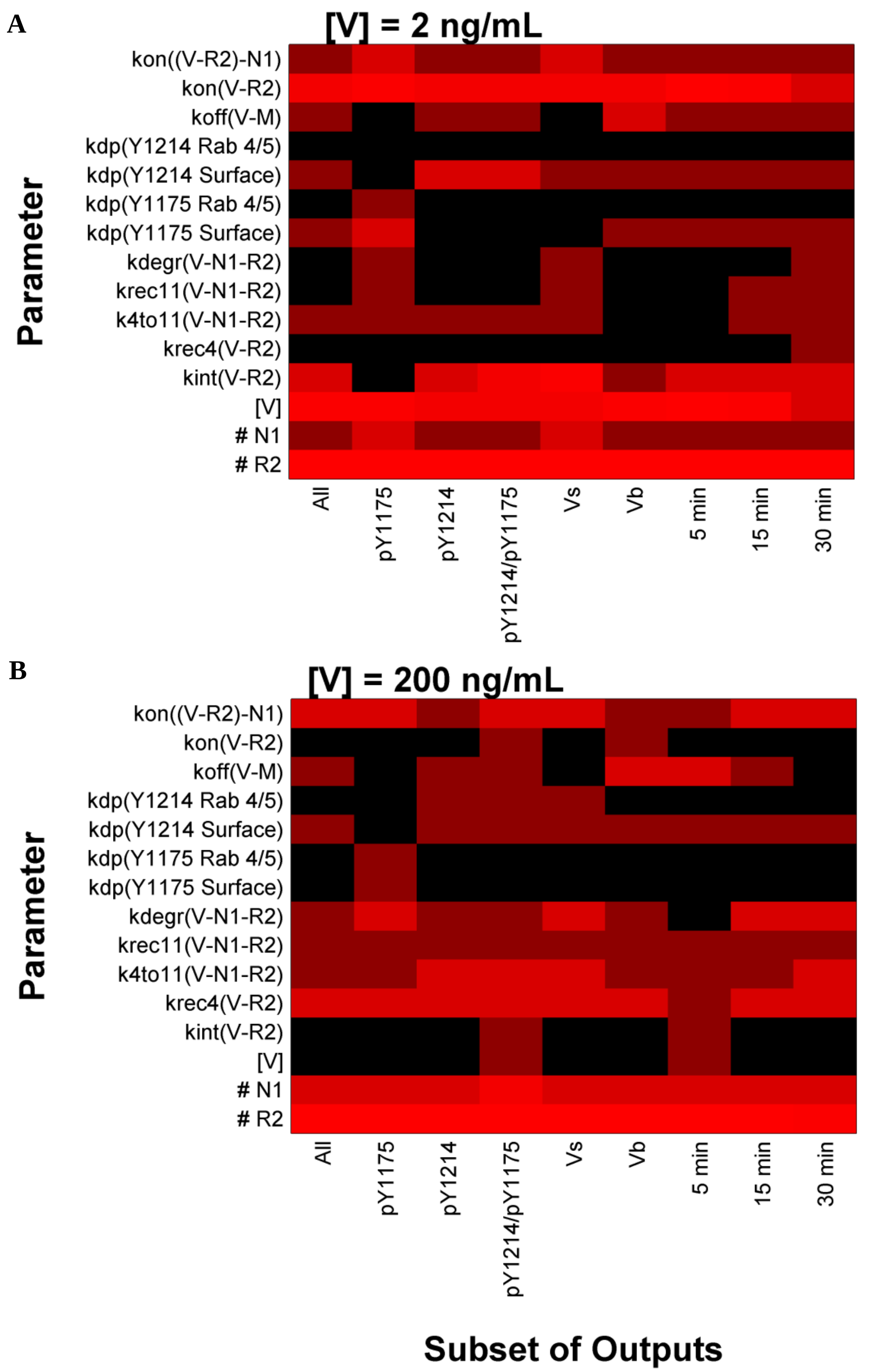

Figure S9. Sensitivity of model outputs varies with VEGF concentration. Sensitivity of outputs to small changes in parameters and initial conditions. Values are average fold change in selected outputs for a 1-fold change in the specified parameter. **A.** [V] = 2 ng/mL. Scale: Black = 0, Bright red (maximum) = 0.77 **B.** [V] = 200 ng/mL. Scale: Black = 0, Bright red = 1.03 (maximum).
