# Supplementary material for: Site-Specific Phosphorylation of VEGFR2 Is Mediated by Receptor Trafficking: Insights from a Computational Model
Source: PLoS Comput Biol. 2015 Jun 12;11(6):e1004158. doi: 10.1371/journal.pcbi.1004158 (PMC4466579; doi:10.1371/journal.pcbi.1004158)
Supplement: S1 Table — (DOCX) [file pcbi.1004158.s010.docx]

**Table S1. Molecules Included in the Model and Simulations**

| Species | Description | Units |
| --- | --- | --- |
| [V] | Free VEGF in extracellular compartment | M |
| [M] | ECM or VEGF-binding sites in extracellular compartment | M |
| [V·M] | Immobilized VEGF (bound to the ECM or surface) | M |
| [R2] | Free VEGFR2 on the cell surface | mol/cm^2^ |
| [V·R2] | Soluble VEGF bound to VEGFR2 on the cell surface | mol/cm^2^ |
| [M$\cdot$V$\cdot$R2] | Matrix-bound VEGF bound to VEGFR2 on the cell surface | mol/cm^2^ |
| [N1] | Free NRP1 on the cell surface | mol/cm^2^ |
| [V·N1] | NRP1-bound VEGF on the cell surface | mol/cm^2^ |
| [V$\cdot$N1$\cdot$R2] | VEGF-NRP1-VEGFR2 complex on the cell surface | mol/cm^2^ |
| [V_rab45_] | Free VEGF in Rab 4/5 endosomes | mol/cm^2^ |
| [R2_rab45_] | VEGFR2 in Rab 4/5 endosomes | mol/cm^2^ |
| [V·R2_rab45_] | V$\cdot$R2 complex in Rab 4/5 endosomes | mol/cm^2^ |
| [N1_rab45_] | Free NRP1 in Rab 4/5 endosomes | mol/cm^2^ |
| [V·N1_rab45_] | NRP1-bound VEGF in Rab 4/5 endosomes | mol/cm^2^ |
| [V·N1·R2_rab45_] | VEGF-NRP1-VEGFR2 complex in Rab 4/5 endosomes | mol/cm^2^ |
| [V_rab11_] | Free VEGF in Rab 11 endosomes | mol/cm^2^ |
| [R2_rab11_] | VEGFR2 in Rab 11 endosomes | mol/cm^2^ |
| [V·R2_rab11_] | V$\cdot$R2 complex in Rab 11 endosomes | mol/cm^2^ |
| [N1_rab11_] | NRP1 in Rab 11 endosomes | mol/cm^2^ |
| [V·N1_rab11_] | NRP1-bound VEGF in Rab 11 endosomes | mol/cm^2^ |
| [V·N1·R2_rab11_] | VEGF-NRP1-VEGFR2 complex in Rab 11 endosomes | mol/cm^2^ |
| [V_deg_] | Degraded VEGF | mol/cm^2^ |
| [R2_deg_] | Degraded VEGFR2 | mol/cm^2^ |
| [V·R2_deg_] | Degraded V$\cdot$R2 | mol/cm^2^ |
| [N1_deg_] | Degraded free NRP1 | mol/cm^2^ |
| [V·N1_deg_] | Degraded NRP1-bound VEGF | mol/cm^2^ |
| [V·N1·R2_deg_] | Degraded VEGF-NRP1-VEGFR2 complex | mol/cm^2^ |

Note: For all reactions, units on surface and internal species are converted to M as needed. Surface species units are mol/cm^2^ of well plate surface area (not cell surface area). Internal species are also in mol/cm^2^ of well plate surface area.
