# Supplementary material for: Site-Specific Phosphorylation of VEGFR2 Is Mediated by Receptor Trafficking: Insights from a Computational Model
Source: PLoS Comput Biol. 2015 Jun 12;11(6):e1004158. doi: 10.1371/journal.pcbi.1004158 (PMC4466579; doi:10.1371/journal.pcbi.1004158)
Supplement: S2 Table — (DOCX) [file pcbi.1004158.s011.docx]

Table S2. Cell Geometry Parameters

| Dimension | Value | Units | Reference |
| --- | --- | --- | --- |
| Cell Surface Area (top only) | 1000 | μm^2^ | Jaffe 1987[1] |
| Cell Volume | 1000 | μm^3^ | Jaffe 1987[1] |
| Total Rab 4/5 Surface Area per Cell | 100 | μm^2^ | Steinman 1976 [2] (assume ½ endosomal surface area) |
| Total Rab 4/5 Volume per Cell | 2.5 | μm^3^ | Griffiths 1989 [3] (assume ½ endosomal volume) |
| Total Rab 11 Surface Area per Cell | 100 | μm^2^ | Steinman 1976 [2] (assume ½ endosomal surface area) |
| Total Rab 11 Volume per Cell | 2.5 | μm^3^ | Griffiths 1989 [3] (assume ½ endosomal volume) |

**References**

1. Jaffe EA. Cell Biology of Endothelial Cells. Human Pathology. 1987;18(3):234-9. doi: 10.1016/s0046-8177(87)80005-9. PubMed PMID: WOS:A1987G487900005.

2. Steinman RM, Brodie SE, Cohn ZA. Membrane Flow during Pinocytosis- Stereologic Analysis. Journal of Cell Biology. 1976;68(3):665-87. doi: 10.1083/jcb.68.3.665. PubMed PMID: WOS:A1976BG95600021.

3. Griffiths G, Back R, Marsh M. A Quantitative Analysis of the Endocytic Pathway in Baby Hamster Kidney Cells. Journal of Cell Biology. 1989;109(6):2703-20. doi: 10.1083/jcb.109.6.2703. PubMed PMID: WOS:A1989CD71100013.
