# Supplementary material for: Site-Specific Phosphorylation of VEGFR2 Is Mediated by Receptor Trafficking: Insights from a Computational Model
Source: PLoS Comput Biol. 2015 Jun 12;11(6):e1004158. doi: 10.1371/journal.pcbi.1004158 (PMC4466579; doi:10.1371/journal.pcbi.1004158)
Supplement: S3 Table — (DOCX) [file pcbi.1004158.s012.docx]

Table S3. Representative Fits to Experimental Trafficking Data [1]

| **Species Measured** | **Location** | **Time**  **(min)** | **Transfected Receptors** | **[VEGF] (ng/mL)** | **Simulation Value** | **Data Value** | **Weight** |
| --- | --- | --- | --- | --- | --- | --- | --- |
| R2 | Rab 4/5 | - | R2 | 0 | **93** | **81** | 1 |
| R2 | Rab 11 | - | R2 | 0 | **6.6** | **2.7** | 1 |
| N1 | Rab 4/5 | - | N1 | 0 | **37** | **48** | 1 |
| N1 | Rab 11 | - | N1 | 0 | **51** | **46** | 1 |
| V·N1·R2 | Rab 4/5 | 30 | R2 + N1 | 50 | **27** | **47** | 1 |
| V·N1·R2 | Rab 11 | 30 | R2 + N1 | 50 | **37** | **45** | 1 |
| V·N1·R2 | Rab 4/5 | 180 | R2 + N1 | 50 | **4.6** | **47** | 1 |
| V·N1·R2 | Rab 11 | 180 | R2 + N1 | 50 | **6.2** | **46** | 1 |
| R2 | Rab 4/5 | 30 | R2 | 50 | **91** | **79** | 1 |
| R2 | Rab 11 | 30 | R2 | 50 | **3.7** | **4.5** | 1 |
| R2 | Rab 4/5 | 180 | R2 | 50 | **66** | **78** | 1 |
| R2 | Rab 11 | 180 | R2 | 50 | **4.8** | **2.6** | 1 |
| N1 | Surface | 30 | R2 + N1 | 50 | **22** | **21** | 1 |
| N1 | Surface | 180 | R2 + N1 | 50 | **7.1** | **90** | 1 |
| R2 | Total | 10 | R2 + N1 | 50 | **38** | **64** | 3 |
| R2 | Total | 30 | R2 + N1 | 50 | **32** | **44** | 3 |
| R2 | Total | 180 | R2 + N1 | 50 | **11** | **33** | 3 |
| N1 | Total | 10 | R2 + N1 | 50 | **91** | **74** | 3 |
| N1 | Total | 30 | R2 + N1 | 50 | **72** | **66** | 3 |
| N1 | Total | 180 | R2 + N1 | 50 | **18** | **45** | 3 |
| % R2 on Surface | | - | R2 + N1 | 0 | **58** | **60** | 5 |

Note: Simulated values and experimental data in Rab4/5 and Rab11 endosomes are expressed as a percentage of the internalized molecule of interest in the indicated compartment. Rab7 data (not shown) is compared to the degraded compartment in the model. Rab7 was not used for parameter fitting, as percentages in the Rab7 compartment are fully specified if the percentages in Rab4/5 and Rab11 endosomes are known. Total values are given as a percentage of the value in unstimulated cells. Receptors included in the count can be free or bound to VEGF.

**References**

1. Ballmer-Hofer K, Andersson AE, Ratcliffe LE, Berger P. Neuropilin-1 promotes VEGFR-2 trafficking through Rab11 vesicles thereby specifying signal output. Blood. 2011;118(3):816-26. doi: 10.1182/blood-2011-01-328773. PubMed PMID: WOS:000292967300045.
