# Supplementary material for: Site-Specific Phosphorylation of VEGFR2 Is Mediated by Receptor Trafficking: Insights from a Computational Model
Source: PLoS Comput Biol. 2015 Jun 12;11(6):e1004158. doi: 10.1371/journal.pcbi.1004158 (PMC4466579; doi:10.1371/journal.pcbi.1004158)
Supplement: S4 Table — (DOCX) [file pcbi.1004158.s013.docx]

**Table S4. Summary of Distribution of Accepted Parameter Sets**

| Parameter | Mean (s^-1^) | Median (s^-1^) | σ (s^-1^) | CV |
| --- | --- | --- | --- | --- |
| **Trafficking** |  |  |  |  |
| k_degr_(V·N1·R2) | 9.26 x 10^-4^ | 6.90 x 10^-4^ | 9.06 x 10^-4^ | 0.98 |
| k_degr_(V·R2) | 2.72 x 10^-5^ | 2.80 x 10^-5^ | 7.58 x 10^-6^ | 0.28 |
| k_degr_(N1) | 1.55 x 10^-4^ | 4.00 x 10^-5^ | 1.92 x 10^-4^ | 1.24 |
| k_rec4_(R2) | 3.73 x 10^-3^ | 3.70 x 10^-3^ | 2.93 x 10^-4^ | 0.08 |
| k_rec11_(N1) | 1.19 x 10^-2^ | 1.30 x 10^-2^ | 2.60 x 10^-3^ | 0.22 |
| k_4to11_(N1) | 1.65 x 10^-2^ | 1.60 x 10^-2^ | 4.18 x 10^-3^ | 0.25 |
|  |  |  |  |  |
| **Phosphorylation** |  |  |  |  |
| k_dp, Y951, surface_ | 4.986 | 0.0027 | 11.9 | 2.39 |
| k_dp, Y1175, surface_ | 7.267 | 3.97 | 8.94 | 1.23 |
| k_dp, Y1214, surface_ | 0.580 | 0.0118 | 1.35 | 2.33 |
| k_dp, Y951, rab45_ | 9.523 | 0.0413 | 25.8 | 2.71 |
| k_dp, Y1175, rab45_ | 0.051 | 0.00172 | 0.184 | 3.61 |
| k_dp, Y1214, rab45_ | 0.041 | 0.00119 | 0.133 | 3.24 |
|  |  |  |  |  |
| Surface/Internal Ratios: k_dp_ | | | | |
| Y951 | 266 | 0.719 | 989 | 3.72 |
| Y1175 | 3440 | 940 | 7750 | 2.25 |
| Y1214 | 64.9 | 6.84 | 190 | 2.93 |
|  |  |  |  |  |
| Y1175/Y1214 | 2760 | 252 | 8810 | 3.19 |

σ: Standard Deviation; CV: coefficient of variation.
