# Supplementary material for: Site-Specific Phosphorylation of VEGFR2 Is Mediated by Receptor Trafficking: Insights from a Computational Model
Source: PLoS Comput Biol. 2015 Jun 12;11(6):e1004158. doi: 10.1371/journal.pcbi.1004158 (PMC4466579; doi:10.1371/journal.pcbi.1004158)
Supplement: S5 Table — (DOCX) [file pcbi.1004158.s014.docx]

Table S5. Summary of Phosphatases Acting on VEGFR2

| Name | Location(s) | Interactions | Residues Dephosphorylated | Residues Not Dephosphorylated | References |
| --- | --- | --- | --- | --- | --- |
| TCPTP (PTPN2) | PM (focal adhesions), N, C | VEGFR2,  α_1_ integrins | Y996, Y1054, Y1059, Y1214 | Y1175 | [1] |
| VEPTP (PTPRB) | PM (cell junctions) | VEGFR2, Tie2, VE-Cadherin | Y951, Y1175 | Y1214 | [2, 3] |
| DEP-1 (CD148, PTPRJ) | PM (cell junctions) | VEGFR2,  VE-Cadherin | Y801, Y951, Y996, Y1054, Y1059, Y1175, Y1214 |  | [4-6] |
| SHP-1 (PTPN6) | PM | VEGFR2, Src, eNOS | Y996, Y1059, Y1175 | Y951 | [7, 8] |
| SHP-2 (PTPN11) | PM | VEGFR2, Tie2, collagen I, D2DR | Y951, Y996, Y1059 | Y1175 | [9-11] |
| PTP1B (PTPN1) | PM/EE, ER | VEGFR2,  VE-cadherin | Y1175 |  | [12-14] |
| PTP-MEG2 (PTPN9) | PM, C (peri-nuclear & vesicular) | VEGFR2 | Y1175 |  | [15] |
| HCPTPA | C | VEGFR2 |  |  | [16] |

PM: Plasma Membrane; N: Nucleus; C: Cytosol; EE: Early Endosomes; ER: endoplasmic reticulum

**References**

1. Mattila E, Auvinen K, Salmi M, Ivaska J. The protein tyrosine phosphatase TCPTP controls VEGFR2 signalling. Journal of Cell Science. 2008;121(21):3570-80. doi: 10.1242/jcs.031898. PubMed PMID: WOS:000260266700010.

2. Mellberg S, Dimberg A, Bahram F, Hayashi M, Rennel E, Ameur A, et al. Transcriptional profiling reveals a critical role for tyrosine phosphatase VE-PTP in regulation of VEGFR2 activity and endothelial cell morphogenesis. FASEB Journal. 2009;23(5):1490-502. doi: 10.1096/fj.08-123810. PubMed PMID: WOS:000266651700025.

3. Hayashi M, Majumdar A, Li X, Adler J, Sun Z, Vertuani S, et al. VE-PTP regulates VEGFR2 activity in stalk cells to establish endothelial cell polarity and lumen formation. Nature Communications. 2013;4. doi: 10.1038/ncomms2683. PubMed PMID: WOS:000318872100029.

4. Chabot C, Spring K, Gratton JP, Elchebly M, Royal I. New Role for the Protein Tyrosine Phosphatase DEP-1 in Akt Activation and Endothelial Cell Survival. Molecular and Cellular Biology. 2009;29(1):241-53. doi: 10.1128/mcb.01374-08. PubMed PMID: WOS:000261697500019.

5. Lampugnani MG, Zanetti A, Corada M, Takahashi T, Balconi G, Breviario F, et al. Contact inhibition of VEGF-induced proliferation requires vascular endothelial cadherin, beta-catenin, and the phosphatase DEP-1/CD148. Journal of Cell Biology. 2003;161(4). doi: 10.1083/jcb.200209019. PubMed PMID: WOS:000183286700015.

6. Lampugnani MG, Orsenigo F, Gagliani MC, Tacchetti C, Dejana E. Vascular endothelial cadherin controls VEGFR-2 internalization and signaling from intracellular compartments. Journal of Cell Biology. 2006;174(4):593-604. doi: 10.1083/jcb.200602080. PubMed PMID: WOS:000239986600017.

7. Bhattacharya R, Kwon J, Wang E, Mukherjee P, Mukhopadhyay D. Src homology 2 (SH2) domain containing protein tyrosine phosphatase-1 (SHP-1) dephosphorylates VEGF Receptor-2 and attenuates endothelial DNA synthesis, but not migration. Journal of molecular signaling. 2008;3:8. doi: 10.1186/1750-2187-3-8. PubMed PMID: MEDLINE:18377662.

8. Cai J, Jiang WG, Ahmed A, Boulton M. Vascular endothelial growth factor-induced endothelial cell proliferation is regulated by interaction between VEGFR-2, SH-PTP1 and eNOS. Microvascular Research. 2006;71(1):20-31. doi: 10.1016/j.mvr.2005.10.004. PubMed PMID: WOS:000235435800003.

9. Sinha S, Vohra PK, Bhattacharya R, Dutta S, Sinha S, Mukhopadhyay D. Dopamine regulates phosphorylation of VEGF receptor 2 by engaging Src-homology-2-domain-containing protein tyrosine phosphatase 2. Journal of Cell Science. 2009;122(18):3385-92. doi: 10.1242/jcs.053124. PubMed PMID: WOS:000269521900021.

10. Mitola S, Brenchio B, Piccinini M, Tertoolen L, Zammataro L, Breier G, et al. Type I collagen limits VEGFR-2 signaling by a SHP2 protein-tyrosine phosphatase-dependent mechanism 1. Circulation Research. 2006;98(1):45-54. doi: 10.1161/01.RES.0000199355.32422.7b. PubMed PMID: WOS:000234419500009.

11. Huang LW, Turck CW, Rao P, Peters KG. Grb2 and SH-PTP2- Potentially Important Endothelial Signaling Molecules Downstream of the TEK/Tie2 Receptor Tyrosine Kinase. Oncogene. 1995;11(10):2097-103. PubMed PMID: WOS:A1995TF29700020.

12. Lanahan A, Zhang X, Fantin A, Zhuang Z, Rivera-Molina F, Speichinger K, et al. The Neuropilin 1 Cytoplasmic Domain Is Required for VEGF-A-Dependent Arteriogenesis. Developmental Cell. 2013;25(2):156-68. doi: 10.1016/j.devcel.2013.03.019. PubMed PMID: WOS:000318327300007.

13. Lanahan AA, Hermans K, Claes F, Kerley-Hamilton JS, Zhuang ZW, Giordano FJ, et al. VEGF Receptor 2 Endocytic Trafficking Regulates Arterial Morphogenesis. Developmental Cell. 2010;18(5):713-24. doi: 10.1016/j.devcel.2010.02.016. PubMed PMID: WOS:000278115500008.

14. Nakamura Y, Patrushev N, Inomata H, Mehta D, Urao N, Kim HW, et al. Role of protein tyrosine phosphatase 1B in vascular endothelial growth factor signaling and cell-cell adhesions in endothelial cells. Circulation Research. 2008;102(10):1182-91. doi: 10.1161/circresaha.107.167080. PubMed PMID: WOS:000256101500009.

15. Hao Q, Samten B, Ji H-L, Zhao ZJ, Tang H. Tyrosine phosphatase PTP-MEG2 negatively regulates vascular endothelial growth factor receptor signaling and function in endothelial cells. American Journal of Physiology-Cell Physiology. 2012;303(5):C548-53. doi: 10.1152/ajpcell.00415.2011. PubMed PMID: WOS:000308466100009.

16. Huang LW, Sankar S, Lin C, Kontos CD, Schroff AD, Cha EH, et al. HCPTPA, a protein tyrosine phosphatase that regulates vascular endothelial growth factor receptor-mediated signal transduction and biological activity. Journal of Biological Chemistry. 1999;274(53):38183-8. doi: 10.1074/jbc.274.53.38183. PubMed PMID: WOS:000084528000091.
