# Supplementary material for: Site-Specific Phosphorylation of VEGFR2 Is Mediated by Receptor Trafficking: Insights from a Computational Model
Source: PLoS Comput Biol. 2015 Jun 12;11(6):e1004158. doi: 10.1371/journal.pcbi.1004158 (PMC4466579; doi:10.1371/journal.pcbi.1004158)
Supplement: S1 Equations — (PDF) [file pcbi.1004158.s015.pdf]

Supplemental Information for:

Site-specific Phosphorylation of VEGFR2 is Mediated by Receptor Trafficking: Insights from a Computational Model

Lindsay Wendel Clegg[a] and Feilim Mac Gabhann[a,b]

[a] Institute for Computational Medicine and Department of Biomedical Engineering, Johns Hopkins University, 3400 N. Charles St., Baltimore MD 21218, USA

[b] Department of Materials Science and Engineering, Johns Hopkins University, 3400 N. Charles St., Baltimore MD 21218, USA

EQUATIONS: Biochemical Reactions and Trafficking Processes

Extracellular Molecular Complexes

Cell Surface Molecular Complexes

Rab 4/5 Molecular Complexes

Rab 11 Molecular Complexes

Degraded Molecular Complexes

EQUATIONS: Biochemical Reactions and Trafficking Processes

Extracellular Molecular Complexes:

$$\frac{d[V]}{dt} = -k_{on,V\cdot M}[V][M] + k_{off,V\cdot M}[V\cdot M] - k_{on,V\cdot R2}[V][R2] + k_{off,V\cdot R2}[V\cdot R2] - k_{on,V\cdot N1}[V][N1] + k_{off,V\cdot N1}[V\cdot N1]$$

$$\frac{d[M]}{dt} = -k_{on,V\cdot M}[V][M] + k_{off,V\cdot M}[V\cdot M] - k_{on,M\cdot(V\cdot R2)}[M][V\cdot R2] + k_{off,M\cdot(V\cdot R2)}[M\cdot V\cdot R2]$$

$$\frac{d[V\cdot M]}{dt} = k_{on,V\cdot M}[V][M] - k_{off,V\cdot M}[V\cdot M] - k_{on,(V\cdot M)\cdot R2}[V\cdot M][R2] + k_{off,(M\cdot V)\cdot R2}[M\cdot V\cdot R2]$$

Cell Surface Molecular Complexes:

$$\frac{d[R2]}{dt} = -k_{on,V\cdot R2}[V][R2] + k_{off,V\cdot R2}[V\cdot R2] - k_{on,(V\cdot M)\cdot R2}[V\cdot M][R2] + k_{off,(M\cdot V)\cdot R2}[M\cdot V\cdot R2] - k_{on,(V\cdot N1)\cdot R2}[V\cdot N1][R2] + k_{off,(V\cdot N1)\cdot R2}[V\cdot N1\cdot R2] - k_{intn,R2}[R2] + k_{rec4,R2}[R2_{rab45}] + k_{rec11,R2}[R2_{rab11}] + s_{R2}$$

$$\frac{d[V\cdot R2]}{dt} = k_{on,V\cdot R2}[V][R2] - k_{off,V\cdot R2}[V\cdot R2] - k_{on,M\cdot(V\cdot R2)}[M][V\cdot R2] + k_{off,M\cdot(V\cdot R2)}[M\cdot V\cdot R2] - k_{on,(V\cdot R2)\cdot N1}[V\cdot R2][N1] + k_{off,(V\cdot R2)\cdot N1}[V\cdot N1\cdot R2] - k_{intn,V\cdot R2}[V\cdot R2] + k_{rec4,V\cdot R2}[(V\cdot R2)_{rab45}] + k_{rec11,V\cdot R2}[(V\cdot R2)_{rab11}]$$

$$\frac{d[V\cdot R2]}{dt} = k_{on,V\cdot R2}[V][R2] - k_{off,V\cdot R2}[V\cdot R2] - k_{on,M\cdot(V\cdot R2)}[M][V\cdot R2] + k_{off,M\cdot(V\cdot R2)}[M\cdot V\cdot R2] - k_{on,(V\cdot R2)\cdot N1}[V\cdot R2][N1] + k_{off,(V\cdot R2)\cdot N1}[V\cdot N1\cdot R2] - k_{intn,V\cdot R2}[V\cdot R2] + k_{rec4,V\cdot R2}[(V\cdot R2)_{rab45}] + k_{rec11,V\cdot R2}[(V\cdot R2)_{rab11}]$$

$$\frac{d\left[M\cdot V\cdot R2\right]}{dt}=k_{on,(V\cdot M)\cdot R2}\left[V\cdot M\right]\left[R2\right]-k_{off,(M\cdot V)\cdot R2}\left[M\cdot V\cdot R2\right]+k_{on,M\cdot(V\cdot R2)}\left[M\right]\left[V\cdot R2\right]$$
$$-k_{off,M\cdot(V\cdot R2)}\left[M\cdot V\cdot R2\right]$$

$$\frac{d\left[N1\right]}{dt}=-k_{on,V\cdot N1}\left[V\right]\left[N1\right]+k_{off,V\cdot N1}\left[V\cdot N1\right]-k_{on,(V\cdot R2)\cdot N1}\left[V\cdot R2\right]\left[N1\right]+k_{off,(V\cdot R2)\cdot N1}\left[V\cdot N1\cdot R2\right]$$
$$-k_{intn,N1}\left[N1\right]+k_{rec4,N1}\left[N1_{rab45}\right]+k_{rec11,N1}\left[N1_{rab11}\right]+s_{N1}$$

$$\frac{d\left[V\cdot N1\right]}{dt}=k_{on,V\cdot N1}\left[V\right]\left[N1\right]-k_{off,V\cdot N1}\left[V\cdot N1\right]-k_{on,R2\cdot(V\cdot N1)}\left[R2\right]\left[V\cdot N1\right]+k_{off,R2\cdot(V\cdot N1)}\left[V\cdot N1\cdot R2\right]$$
$$-k_{intn,V\cdot N1}\left[V\cdot N1\right]+k_{rec4,V\cdot N1}\left[(V\cdot N1)_{rab45}\right]+k_{rec11,V\cdot N1}\left[(V\cdot N1)_{rab11}\right]$$

$$\frac{d\left[V\cdot N1\cdot R2\right]}{dt}=k_{on,(V\cdot N1)\cdot R2}\left[V\cdot N1\right]\left[R2\right]-k_{off,(V\cdot N1)\cdot R2}\left[V\cdot N1\cdot R2\right]+k_{on,(V\cdot R2)\cdot N1}\left[V\cdot R2\right]\left[N1\right]$$
$$-k_{off,(V\cdot R2)\cdot N1}\left[V\cdot N1\cdot R2\right]-k_{intn,V\cdot N1\cdot R2}\left[V\cdot N1\cdot R2\right]+k_{rec4,V\cdot N1\cdot R2}\left[(V\cdot N1\cdot R2)_{rab45}\right]$$
$$+k_{rec11,V\cdot N1\cdot R2}\left[(V\cdot N1\cdot R2)_{rab11}\right]$$

Rab 4/5 Molecular Complexes:

$$\frac{d\left[R2_{rab45}\right]}{dt}=-k_{on,V\cdot R2_{rab45}}\left[V_{rab45}\right]\left[R2_{rab45}\right]+k_{off,V\cdot R2_{rab45}}\left[\left(V\cdot R2\right)_{rab45}\right]$$
$$-k_{on,(V\cdot N1)\cdot R2_{rab45}}\left[\left(V\cdot N1\right)_{rab45}\right]\left[R2_{rab45}\right]+k_{off,(V\cdot N1)\cdot R2_{rab45}}\left[\left(V\cdot N1\cdot R2\right)_{rab45}\right]+k_{intn,R2}\left[R2\right]$$
$$-k_{rec4,R2_{rab45}}\left[R2_{rab45}\right]-k_{4to11,R2_{rab45}}\left[R2_{rab45}\right]-k_{degr,R2_{rab45}}\left[R2_{rab45}\right]$$

$$\frac{d\left[\left(V\cdot R2\right)_{rab45}\right]}{dt}=k_{on,V\cdot R2_{rab45}}\left[V_{rab45}\right]\left[R2_{rab45}\right]-k_{off,V\cdot R2_{rab45}}\left[\left(V\cdot R2\right)_{rab45}\right]$$
$$-k_{on,(V\cdot R2)\cdot N1_{rab45}}\left[\left(V\cdot R2\right)_{rab45}\right]\left[N1_{rab45}\right]+k_{off,(V\cdot R2)\cdot N1_{rab45}}\left[\left(V\cdot N1\cdot R2\right)_{rab45}\right]$$
$$+k_{intn,V\cdot R2}\left[V\cdot R2\right]-k_{rec4,V\cdot R2_{rab45}}\left[(V\cdot R2)_{rab45}\right]-k_{4to11,4,V\cdot R2_{rab45}}\left[\left(V\cdot R2\right)_{rab45}\right]$$
$$-k_{degr,V\cdot R2_{rab45}}\left[\left(V\cdot R2\right)_{rab45}\right]$$

$$\frac{d\left[V_{rab45}\right]}{dt} = -k_{on,V\cdot R2_{rab45}}\left[V_{rab45}\right]\left[R2_{rab45}\right] + k_{off,V\cdot R2_{rab45}}\left[\left(V\cdot R2\right)_{rab45}\right] - k_{on,V\cdot N1_{rab45}}\left[V_{rab45}\right]\left[N1_{rab45}\right]$$
$$+k_{off,V\cdot N1_{rab45}}\left[\left(V\cdot N1\right)_{rab45}\right] - k_{degr,V_{rab45}}\left[V_{rab45}\right]$$

$$\frac{d\left[N1_{rab45}\right]}{dt} = -k_{on,V\cdot N1_{rab45}}\left[V_{rab45}\right]\left[N1_{rab45}\right] + k_{off,V\cdot N1_{rab45}}\left[\left(V\cdot N1\right)_{rab45}\right]$$
$$-k_{on,(V\cdot R2)\cdot N1_{rab45}}\left[\left(V\cdot R2\right)_{rab45}\right]\left[N1_{rab45}\right] + k_{off,(V\cdot R2)\cdot N1_{rab45}}\left[\left(V\cdot N1\cdot R2\right)_{rab45}\right] + k_{intn,N1}\left[N1\right]$$
$$-k_{rec4,N1_{rab45}}\left[N1_{rab45}\right] - k_{4to11,N1_{rab45}}\left[N1_{rab45}\right] - k_{degr,N1_{rab45}}\left[N1_{rab45}\right]$$

$$\frac{d\left[\left(V\cdot N1\right)_{rab45}\right]}{dt} = k_{on,V\cdot N1_{rab45}}\left[V_{rab45}\right]\left[N1_{rab45}\right] - k_{off,V\cdot N1_{rab45}}\left[\left(V\cdot N1\right)_{rab45}\right]$$
$$-k_{on,(V\cdot N1)\cdot R2_{rab45}}\left[\left(V\cdot N1\right)_{rab45}\right]\left[R2_{rab45}\right] + k_{off,(V\cdot N1)\cdot R2_{rab45}}\left[\left(V\cdot N1\cdot R2\right)_{rab45}\right]$$
$$+k_{intn,V\cdot N1}\left[V\cdot N1\right] - k_{rec4,V\cdot N1_{rab45}}\left[\left(V\cdot N1\right)_{rab45}\right] - k_{4to11,V\cdot N1_{rab45}}\left[\left(V\cdot N1\right)_{rab45}\right]$$
$$-k_{degr,V\cdot N1_{rab45}}\left[\left(V\cdot N1\right)_{rab45}\right]$$

$$\frac{d\left[\left(V\cdot N1\cdot R2\right)_{rab45}\right]}{dt} = k_{on,(V\cdot R2)\cdot N1_{rab45}}\left[\left(V\cdot R2\right)_{rab45}\right]\left[N1_{rab45}\right] - k_{off,(V\cdot R2)\cdot N1_{rab45}}\left[\left(V\cdot N1\cdot R2\right)_{rab45}\right]$$
$$+k_{on,(V\cdot N1)\cdot R2_{rab45}}\left[\left(V\cdot N1\right)_{rab45}\right]\left[R2_{rab45}\right] - k_{off,(V\cdot N1)\cdot R2_{rab45}}\left[\left(V\cdot N1\cdot R2\right)_{rab45}\right]$$
$$+k_{intn,V\cdot N1\cdot R2}\left[V\cdot N1\cdot R2\right] - k_{rec4,V\cdot N1\cdot R2_{rab45}}\left[\left(V\cdot N1\cdot R2\right)_{rab45}\right]$$
$$-k_{4to11,V\cdot N1\cdot R2_{rab45}}\left[\left(V\cdot N1\cdot R2\right)_{rab45}\right] - k_{degr,V\cdot N1\cdot R2_{rab45}}\left[\left(V\cdot N1\cdot R2\right)_{rab45}\right]$$

Rab 11 Molecular Complexes:

$$\frac{d\left[R2_{rab11}\right]}{dt} = -k_{on,V\cdot R2_{rab11}}\left[V_{rab11}\right]\left[R2_{rab11}\right] + k_{off,V\cdot R2_{rab11}}\left[\left(V\cdot R2\right)_{rab11}\right]$$
$$-k_{on,(V\cdot N1)\cdot R2_{rab11}}\left[\left(V\cdot N1\right)_{rab11}\right]\left[R2_{rab11}\right] + k_{off,(V\cdot N1)\cdot R2_{rab11}}\left[\left(V\cdot N1\cdot R2\right)_{rab11}\right]$$
$$+k_{4to11,R2_{rab45}}\left[R2_{rab45}\right] - k_{rec11,R2_{rab11}}\left[R2_{rab11}\right]$$

$$\frac{d\left[(V\cdot R2)_{rab11}\right]}{dt}=k_{on,V\cdot R2_{rab11}}\left[V_{rab11}\right]\left[R2_{rab11}\right]-k_{off,V\cdot R2_{rab11}}\left[(V\cdot R2)_{rab11}\right]$$
$$-k_{on,(V\cdot R2)\cdot N1_{rab11}}\left[(V\cdot R2)_{rab11}\right]\left[N1_{rab11}\right]+k_{off,(V\cdot R2)\cdot N1_{rab11}}\left[(V\cdot N1\cdot R2)_{rab11}\right]$$
$$+k_{4to11,V\cdot R2_{rab45}}\left[(V\cdot R2)_{rab45}\right]-k_{rec11,V\cdot R2_{rab11}}\left[(V\cdot R2)_{rab11}\right]$$

$$\frac{d\left[V_{rab11}\right]}{dt}=-k_{on,V\cdot R2_{rab11}}\left[V_{rab11}\right]\left[R2_{rab11}\right]+k_{off,V\cdot R2_{rab11}}\left[(V\cdot R2)_{rab11}\right]-k_{on,V\cdot N1_{rab11}}\left[V_{rab11}\right]\left[N1_{rab11}\right]$$
$$+k_{off,V\cdot N1_{rab11}}\left[(V\cdot N1)_{rab11}\right]$$

$$\frac{d\left[N1_{rab11}\right]}{dt}=-k_{on,V\cdot N1_{rab11}}\left[V_{rab11}\right]\left[N1_{rab11}\right]+k_{off,V\cdot N1_{rab11}}\left[(V\cdot N1)_{rab11}\right]$$
$$-k_{on,(V\cdot R2)\cdot N1_{rab11}}\left[(V\cdot R2)_{rab11}\right]\left[N1_{rab11}\right]+k_{off,(V\cdot R2)\cdot N1_{rab11}}\left[(V\cdot N1\cdot R2)_{rab11}\right]$$
$$+k_{4to11,N1_{rab45}}\left[N1_{rab45}\right]-k_{rec11,N1_{rab11}}\left[N1_{rab11}\right]$$

$$\frac{d\left[(V\cdot N1)_{rab11}\right]}{dt}=k_{on,V\cdot N1_{rab11}}\left[V_{rab11}\right]\left[N1_{rab11}\right]-k_{off,V\cdot N1_{rab11}}\left[(V\cdot N1)_{rab11}\right]$$
$$-k_{on,(V\cdot N1)\cdot R2_{rab11}}\left[(V\cdot N1)_{rab11}\right]\left[R2_{rab11}\right]+k_{off,(V\cdot N1)\cdot R2_{rab11}}\left[(V\cdot N1\cdot R2)_{rab11}\right]$$
$$+k_{4to11,V\cdot N1_{rab45}}\left[(V\cdot N1)_{rab45}\right]-k_{rec11,V\cdot N1_{rab11}}\left[(V\cdot N1)_{rab11}\right]$$

$$\frac{d\left[(V\cdot N1\cdot R2)_{rab11}\right]}{dt}=k_{on,(V\cdot R2)\cdot N1_{rab11}}\left[(V\cdot R2)_{rab11}\right]\left[N1_{rab11}\right]-k_{off,(V\cdot R2)\cdot N1_{rab11}}\left[(V\cdot N1\cdot R2)_{rab11}\right]$$
$$+k_{on,(V\cdot N1)\cdot R2_{rab11}}\left[(V\cdot N1)_{rab11}\right]\left[R2_{rab11}\right]-k_{off,(V\cdot N1)\cdot R2_{rab11}}\left[(V\cdot N1\cdot R2)_{rab11}\right]$$
$$+k_{4to11,V\cdot N1\cdot R2_{rab45}}\left[(V\cdot N1\cdot R2)_{rab45}\right]-k_{rec11,V\cdot N1\cdot R2_{rab11}}\left[(V\cdot N1\cdot R2)_{rab11}\right]$$

Degraded Molecular Complexes:

$$\frac{d\left[R2_{deg}\right]}{dt}=k_{degr,R2_{rab45}}\left[R2_{rab45}\right]$$

$$\frac{d\left[(V\cdot R2)_{deg}\right]}{dt}=k_{degr,V\cdot R2_{rab45}}\left[(V\cdot R2)_{rab45}\right]$$

$$\frac{d\left[V_{deg}\right]}{dt} = k_{degr,V_{rab45}}[V_{rab45}]$$

$$\frac{d\left[N1_{deg}\right]}{dt} = k_{degr,N1_{rab45}}[N1_{rab45}]$$

$$\frac{d\left[\left(V \cdot N1\right)_{deg}\right]}{dt} = k_{degr,V \cdot N1_{rab45}}[(V \cdot N1)_{rab45}]$$

$$\frac{d\left[\left(V \cdot N1 \cdot R2\right)_{deg}\right]}{dt} = k_{degr,V \cdot N1 \cdot R2_{rab45}}[(V \cdot N1 \cdot R2)_{rab45}]$$
